# Serine Proteolytic Pathway Activation Reveals an Expanded Ensemble of Wound Response Genes in *Drosophila*

**DOI:** 10.1371/journal.pone.0061773

**Published:** 2013-04-24

**Authors:** Rachel A. Patterson, Michelle T. Juarez, Anita Hermann, Roman Sasik, Gary Hardiman, William McGinnis

**Affiliations:** 1 Section of Cell and Developmental Biology, University of California San Diego, La Jolla, California, United States of America; 2 Sophie Davis School of Biomedical Education, City College New York, New York, New York, United States of America; 3 Department of Medicine, University of California San Diego, La Jolla, California, United States of America; Stockholm University, Sweden

## Abstract

After injury to the animal epidermis, a variety of genes are transcriptionally activated in nearby cells to regenerate the missing cells and facilitate barrier repair. The range and types of diffusible wound signals that are produced by damaged epidermis and function to activate repair genes during epidermal regeneration remains a subject of very active study in many animals. In *Drosophila* embryos, we have discovered that serine protease function is locally activated around wound sites, and is also required for localized activation of epidermal repair genes. The serine protease trypsin is sufficient to induce a striking global epidermal wound response without inflicting cell death or compromising the integrity of the epithelial barrier. We developed a trypsin wounding treatment as an amplification tool to more fully understand the changes in the *Drosophila* transcriptome that occur after epidermal injury. By comparing our array results with similar results on mammalian skin wounding we can see which evolutionarily conserved pathways are activated after epidermal wounding in very diverse animals. Our innovative serine protease-mediated wounding protocol allowed us to identify 8 additional genes that are activated in epidermal cells in the immediate vicinity of puncture wounds, and the functions of many of these genes suggest novel genetic pathways that may control epidermal wound repair. Additionally, our data augments the evidence that clean puncture wounding can mount a powerful innate immune transcriptional response, with different innate immune genes being activated in an interesting variety of ways. These include puncture-induced activation only in epidermal cells in the immediate vicinity of wounds, or in all epidermal cells, or specifically in the fat body, or in multiple tissues.

## Introduction

*Drosophila*’s epithelial barriers provide an organismal shield from physical damage and microbial infection. In *Drosophila*, the epidermal barrier consists of a single cell layer that secretes an impermeable, multilayered cuticle at the apical surface. The strength and impermeability of the cuticle are achieved partly through the cross-linking of protein and chitin polymers by reactive quinones [1]. In mammals, the epidermis consists of several layers, the outermost being the stratum corneum, which is composed of dead squamous epithelial cells encased in a cornified cellular envelope, analogous to the *Drosophila* cuticle [2]. Although *Drosophila* and mammalian skin are structurally different, some of the genes that control the formation and repair of epidermal barriers are evolutionarily conserved between *Drosophila* and mammals, making *Drosophila* an advantageous model organism for studying the process of epidermal wound healing [3]–[5]. For example, the *grainy head* (*grh*) gene encodes a conserved transcriptional regulator of epidermal barrier regeneration in both *Drosophila* and mammals [3], [6]–[9]. Additionally, many components of the Jun N-terminal kinase (JNK) signaling cascade, leading to the activation of the AP-1 transcription factor (*Jun/Fos*), promote epidermal wound closure in diverse animal phyla [10]–[13].

At present we know only 10 genes that are transcriptionally activated in a localized zone of epidermal cells around clean puncture or laser wounds in late-stage *Drosophila* embryos [6], [7], [14]–[17]. Some of these genes are directly involved in cuticle regeneration/remodeling, like the genes that encode the enzymes dopa decarboxylase (*Ddc)*, transglutaminase 1 *(TGM1*), tyrosine hydroxylase *(ple)*, and chitin synthase (*kkv*) [18]. Other locally activated wound response genes are involved in re-epithelialization, like *misshapen* (*msn*), which encodes a JNK kinase kinase kinase, and *stitcher (stit)* which encodes a receptor tyrosine kinase (RTK) and *chickadee* which encodes an actin recycling filament protein [6], [10], [11], [16], [17]. Additional locally activated wound genes most likely function to transduce wound signals or limit their spread. These include the aforementioned *stitcher*; *Gadd45*, a gene involved in growth arrest and MAP kinase pathway regulation [15], as well as two other genes, *Flotillin-2 (Flo-2*) and *Src42A*, that function to restrict the spread of local wound signals [14]. We developed fluorescent reporter genes driven by wound-induced transcriptional enhancers from some of the genes mentioned above, examples being *Ddc* and *ple* wound reporters [6], [7] (Materials and Methods).

We know about some of the signaling molecules and transcription factors that either activate or restrict the expression of genes that repair the *Drosophila* epidermal barrier. For example, the transcription factor Grainy head is directly regulated by extracellular signal-regulated kinase (ERK) phosphorylation, and is required for wound-induced activation of *stit*, *Ddc* and *msn* in embryonic epidermal cells [16], [19]. The *stitcher* gene, which encodes a Ret-family RTK, is required for robust induction of ERK phosphorylation around wound sites, and is also required for robust activation of *Ddc* and *ple* transcription around epidermal wound sites [16]. However, the signal responsible for activating *stitcher* remains a mystery [16]. Another RTK, *PDGF- and VEGF-receptor related* (*Pvr*), and one of its ligands, *Pvf1*, are required for epidermal cell migration to close wound gaps in *Drosophila* larval epidermis [20]. Also, another RTK, *EGFR*, regulates epidermal wound re-epithelialization, since *EGFR* mutants display a much higher frequency of open wounds compared to wild-type *Drosophila* embryos [21]. In summary, wound healing is a complex biological process that requires the orchestrated cooperation of ERK, *grh*, and at least two RTK signaling pathways, in addition to other unidentified pathways, to fully regenerate coherent epithelial and cuticular barriers.

Some diffusible signals are used during wound repair in both vertebrates and *Drosophila*. For example, wounded zebrafish tails quickly establish a hydrogen peroxide (H_2_O_2_) gradient that is required to attract neutrophils to wound sites [22]. The evolutionarily conserved enzyme that is activated to produce the H_2_O_2_ gradient is *Dual oxidase* (*Duox*), and in *Drosophila* this enzyme is required for hemocyte recruitment to embryonic wounds sites [23]. *Duox* is also required for the activation of epidermal wound reporter genes surrounding wound sites in the *Drosophila* epidermis [14]. Serine protease function is believed to act downstream of *Duox*, as trypsin-wounded *Duox* mutants exhibit global *ple* wound reporter gene expression [14].

Our aim was to establish a broader understanding of the genome-wide transcriptional response at different time points in the epidermis around clean puncture wounds. There is a signal/background problem with either puncture or laser-wounded embryos in conjunction with microarray technology because only a small subset of *Drosophila* epidermal cells exhibit activation of localized wound regeneration genes [15]. To combat this predicament, we developed a protocol that takes advantage of trypsin-mediated wounding in conjunction with microarray technology to determine changes in the transcriptome of wounded embryos.

In this paper we show that endogenous serine protease activity is localized around wound sites, and that serine protease activity is required for the activation of epidermal wound genes. Exogenously supplied trypsin, which apparently mimics the function of endogenous serine proteases, can globally activate epidermal wound reporter genes without damaging the integrity of epidermal cell junctions or inducing high levels of cellular death. We find that trypsin activates epidermal wound response gene expression in a manner dependent on *grainy head*, which puts serine protease activity in the context of a known wound gene activation pathway [14]. Our comparison of the wound transcriptome of *Drosophila* to that observed in mammals indicates that many common regulatory genes are upregulated in both animals after epidermal wounding. Our trypsin-amplified wounding protocol, followed by *in situ* hybridization, allowed us to identify 8 new wound response genes that are locally-activated in the epidermis, nearly doubling the number of previously reported epidermal wound response genes. Furthermore, our data shows that clean puncture wounding can mount a robust innate immune transcriptional response both locally and globally in the epidermis, as well as in the fat body, in a manner that depends on the specific response gene.

## Results

### Serine Protease Activity is Required to Activate Epidermal Wound Reporter Genes

**Figure 1:**
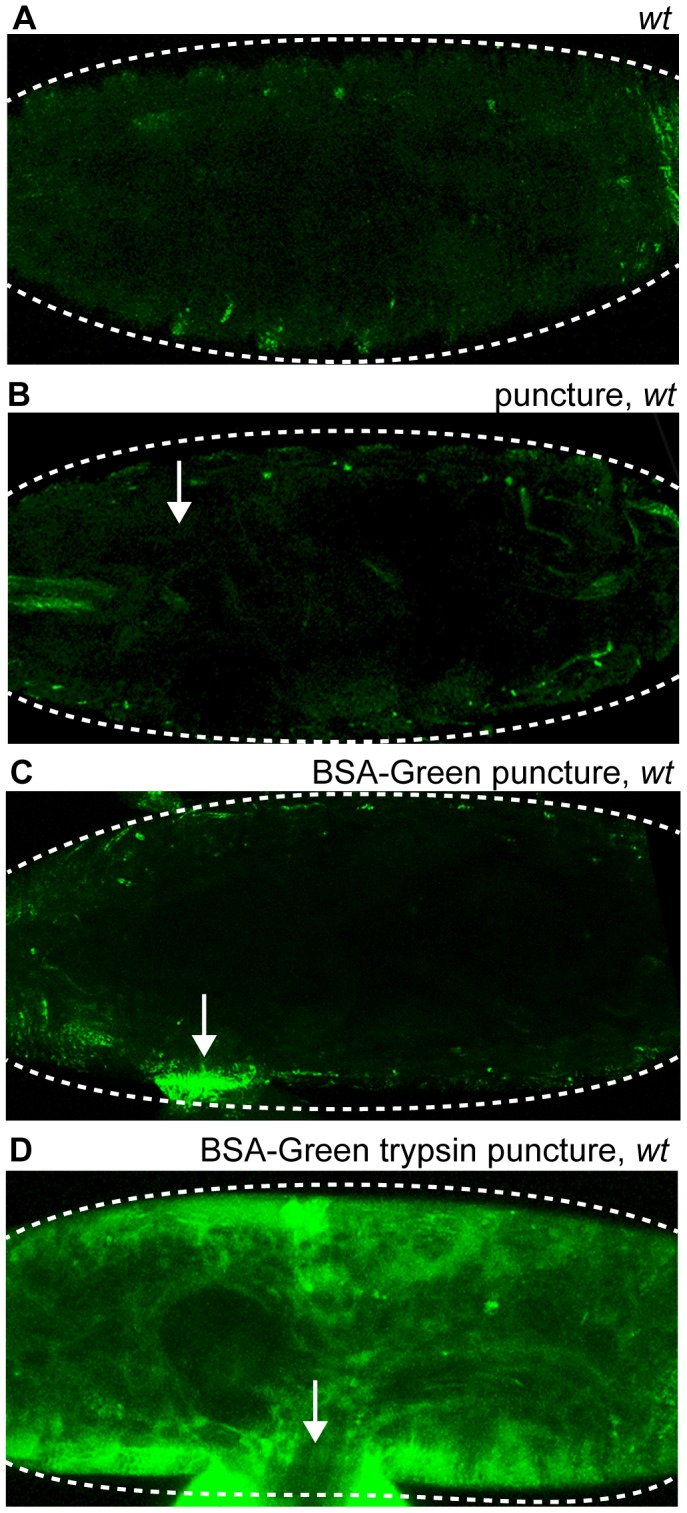
Localized endogenous proteolytic activity occurs at clean puncture wound sites. Confocal images of Bovine Serum Albumin conjugated-Green (BSA-Green) wounded wild-type embryos. (A) Unwounded wild-type embryos display no fluorescence. (B) Puncture wounded wild-type embryos display no fluorescence at the wound site. (C) Wild-type embryos puncture wounded with BSA-Green exhibit green fluorescence surrounding wound site at 30 minutes after wounding, indicating proteolysis of BSA. (D) Simultaneous puncture wounding of trypsin along with BSA-Green results in whole body cavity green fluorescence 30 minutes after wounding. Arrows mark the wound site. Dashed lines in the data panels mark the outlines of embryos.

Serine protease cascades and proteolytic processing of receptor ligands are required for the activation of important localized or systemic signaling pathways that control arthropod dorsoventral polarity, innate immunity, coagulation, and melanization [24]–[30]. We hypothesized that the signaling pathways that activate transcription around *Drosophila* epidermal wound sites might also be dependent on protease activity. To assess endogenous proteolytic activity we used bovine serum albumin conjugated to a quenched fluorescent dye (BSA-Green, Molecular Probes) that emits a signal after proteolytic degradation of the BSA substrate. Wild-type stage 15–17 *Drosophila* embryos that were puncture wounded with BSA-Green showed fluorescent signals localized around wound sites in contrast to wild-type or wounded wild-type embryos at the same developmental stage (Figure 1A, B, C). As a positive control for BSA-Green proteolysis, wild-type embryos that were puncture wounded with BSA-Green pre-incubated with trypsin, showed fluorescent signal throughout the entire embryonic body cavity (Figure 1D). These results reveal that localized endogenous proteolytic activity occurs around clean puncture wound sites.

**Figure 2:**
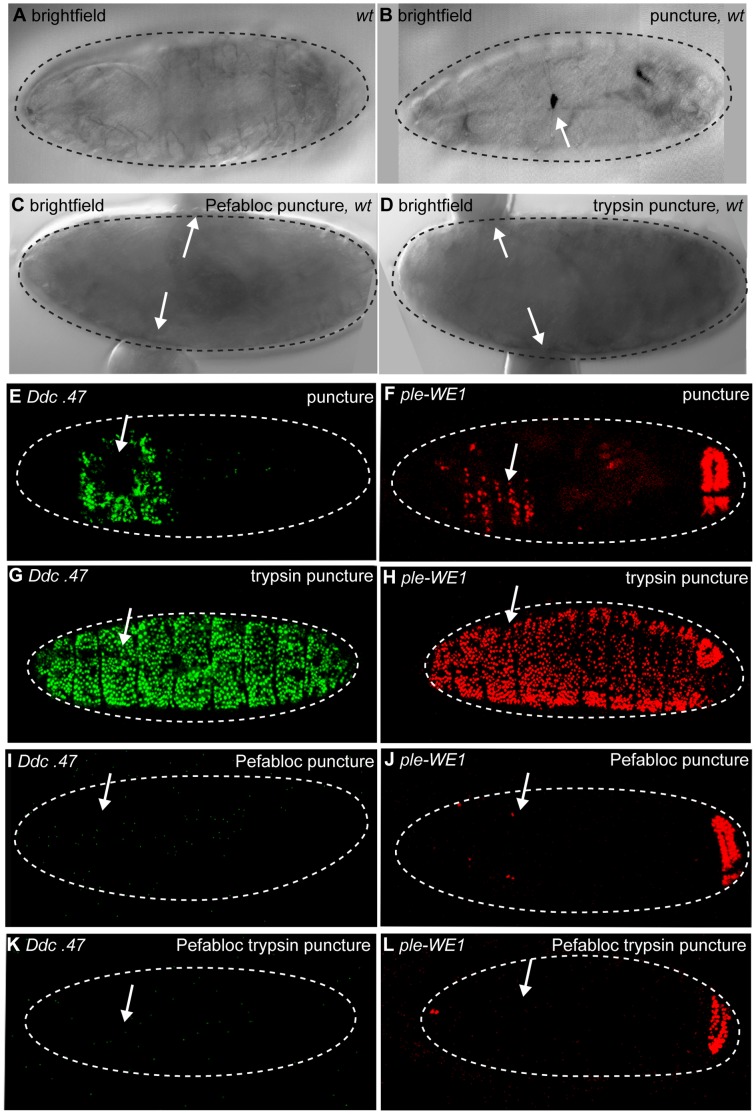
Serine proteases are required and sufficient for *Ddc*.47 and *ple*-WE1 activation. Bright field images of wild-type stage 15–17 embryos. *Ddc*.47 and *ple*-WE1 are fluorescent reporters that include wound-induced DNA enhancers from the *Ddc* and *ple* loci, respectively. (A, C, D) A melanized wound site is not observed in unwounded, Pefabloc wounded, or trypsin wounded embryos. (B) Melanization at the wound site occurs after puncture-only wounding of wild-type embryos. Confocal images of *Ddc*.47 and *ple*-WE1 embryos 6 hours post wounding. (E, F) Control puncture, water puncture, and HCl (trypsin buffer) puncture wounded *Ddc*.47 and *ple*-WE1 embryos all exhibit localized reporter activation at epidermal wound sites. (G, H) Trypsin puncture wounded *Ddc*.47 and *ple-*WE1 embryos exhibit global reporter activation. (I, J) Pefabloc puncture wounded *Ddc*.47 and *ple*-WE1 embryos do not activate reporter at the wound site. (K, L) Pefabloc trypsin puncture wounded *Ddc*.47 and *ple*-WE1 embryos do not activate any epidermal wound reporter expression. The anal pad expression provided by the enhancer in the *ple*-WE1 wound reporter controls for developmental stage. Arrows mark the wound site. Dashed lines in the data panels mark the outlines of embryos.

Since wound response transcripts accumulate in roughly the same localized epidermal region [6] as the observed BSA-Green signal, we tested whether serine protease activity was sufficient to induce epidermal wound reporter genes. To do this, we puncture wounded late stage *Drosophila* embryos with a trypsin-filled needle to monitor wound-dependent activation of *Ddc* and *ple* wound reporter genes [6], [7]. Remarkably, puncture wounding with trypsin, a serine proteinase in the trypsin/chymotrypsin family, resulted in a dramatic global activation of the epidermal wound reporters, while puncture wounding with needles filled with carrier solution gave the typical localized reporter activity (Figure 2G, H, E, F). Similar global activation of the *Ddc* epidermal wound reporter gene was seen after wounding with Proteinase K, another serine protease in the subtilisin family (Figure S1A, B). In contrast, puncture wounding embryos with papain, a cysteine protease, resulted in localized epidermal activation of wound reporters around wound sites plus weak, patchy reporter activation elsewhere (Figure S1C, D). Embryos puncture wounded with Marimastat (Tocris), a broad spectrum matrix metalloproteinase inhibitor [31], still activated the *Ddc* wound reporter surrounding epidermal wound sites, suggesting that matrix metalloproteinase functions are not required to activate epidermal wound reporter genes (Figure S1E, F). Taken together, these results indicate that serine proteases are sufficient to induce global wound response reporter expression, and that the serine proteinase family can function with some specificity in activating wound reporters.

Stein and Nüsslein-Volhard [26] used a serine protease inhibitor to test whether Toll-dependent dorsal-ventral signaling was dependent on serine protease activity in early *Drosophila* embryos. We tested whether localized wound reporter activation was serine protease-dependent by puncture wounding late stage *Drosophila* embryos with needles filled with the specific and irreversible serine protease inhibitor Pefabloc [32]. Puncture wounding of embryos with Pefabloc resulted in complete inhibition of *Ddc* and *ple* reporter gene activation around wound sites compared to control embryos wounded with carrier solution (Figure 2I, J, E, F). Since Pefabloc might simply arrest development, we capitalized on the fact that the transgene with the *ple* wound reporter gene shows wound-independent reporter expression in anal pads from stage 15 until larval hatching. This anal pad reporter expression was unaffected in Pefabloc puncture wounded embryos, indicating that Pefabloc treated embryos progress developmentally for at least the 5 hour period we observed post-wounding (Figure 2J). We also tested whether non-protease contaminants in the trypsin solutions might activate wound reporter genes by wounding with a mixture of Pefabloc and trypsin, and saw no wound-dependent epidermal reporter activity, indicating that the global reporter activation seen after trypsin wounding can be attributed specifically to trypsin function (Figure 2K, L). In summary, our results suggest that serine protease function is both sufficient and required for activation of both epidermal wound reporters.

### Characterization of Trypsin Treatment in Late-stage *Drosophila* Embryos

Trypsin treatment did not detectably diminish the ability of epidermal cells to activate wound reporter genes over a period of hours, and visual observations of Fasciclin III (FasIII) staining indicated that trypsin-treated epidermis had a morphology indistinguishable from untreated epidermis (Figure S2A, B). However we noticed that very high concentrations of trypsin resulted in a significant level of organismal death before larval hatching (Table S1). Therefore, we did additional control experiments to test whether the concentration of trypsin we used was causing paracellular barrier defects, or increased cell death, in the embryonic epidermis.

**Figure 3:**
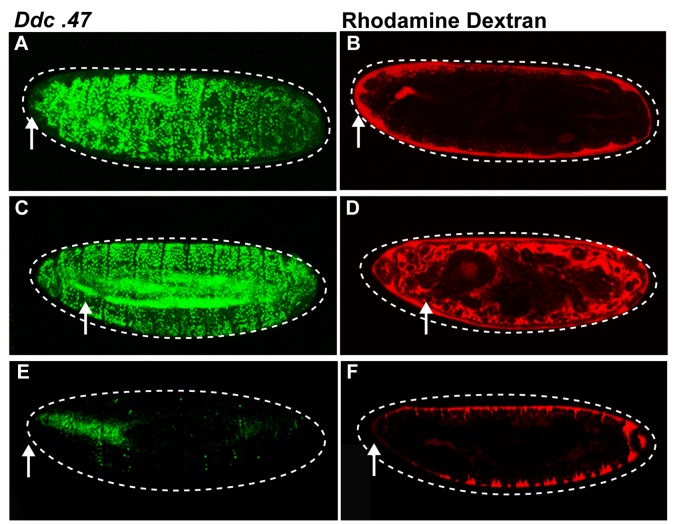
Trypsin treatment does not compromise epidermal barrier integrity. *Ddc*.47 is a fluorescent reporter that includes a wound-induced DNA enhancer from the *Ddc* locus. Confocal images of *Ddc*. 47 (green) embryos injected with fluorescent Rhodamine Dextran (red) to assess epidermal integrity and reporter activation after trypsin puncture wounding. (A, B) Perivitelline injection of trypsin along with Rhodamine Dextran globally activates the *Ddc*.47 wound reporter without compromising the epidermal barrier since Rhodamine Dextran is limited to the perivitelline space. (C, D) Embryos punctured with trypsin and Rhodamine Dextran globally activate *Ddc*.47 wound reporter, but epidermal integrity is lost as Rhodamine Dextran is observed within the embryonic body cavity. (E, F) Control embryos that have been injected in the perivitelline space with Rhodamine Dextran in carrier solution do not activate the *Ddc*.47 wound reporter. Arrows mark the wound site. Dashed lines in the data panels mark the outlines of embryos.

To test whether our trypsin treatment was compromising the epidermal paracellular barrier, we injected trypsin into the perivitelline space. This allowed trypsin to access only the apical side of epidermal cells, which at stage 15–16 have a developing cuticle barrier on the apical surface. The presence of trypsin only on the apical side of epidermal cells was sufficient to activate widespread epidermal wound reporter activity (Figure 3A), and was not associated with a detectable breach in the epidermal paracellular barrier, since Rhodamine Dextran in the perivitelline space did not enter the body cavity, even after hours of trypsin treatment (Figure 3B). Control punctures with Rhodamine Dextran showed that the dye can fluoresce in the body cavity (Figure 3C, D). Injection of Rhodamine Dextran alone into the perivitelline space did not activate wound reporters (Figure 3E, F). Thus, the widespread activation of wound reporters induced by trypsin treatment is not due to compromised epidermal barrier integrity.

**Figure 4:**
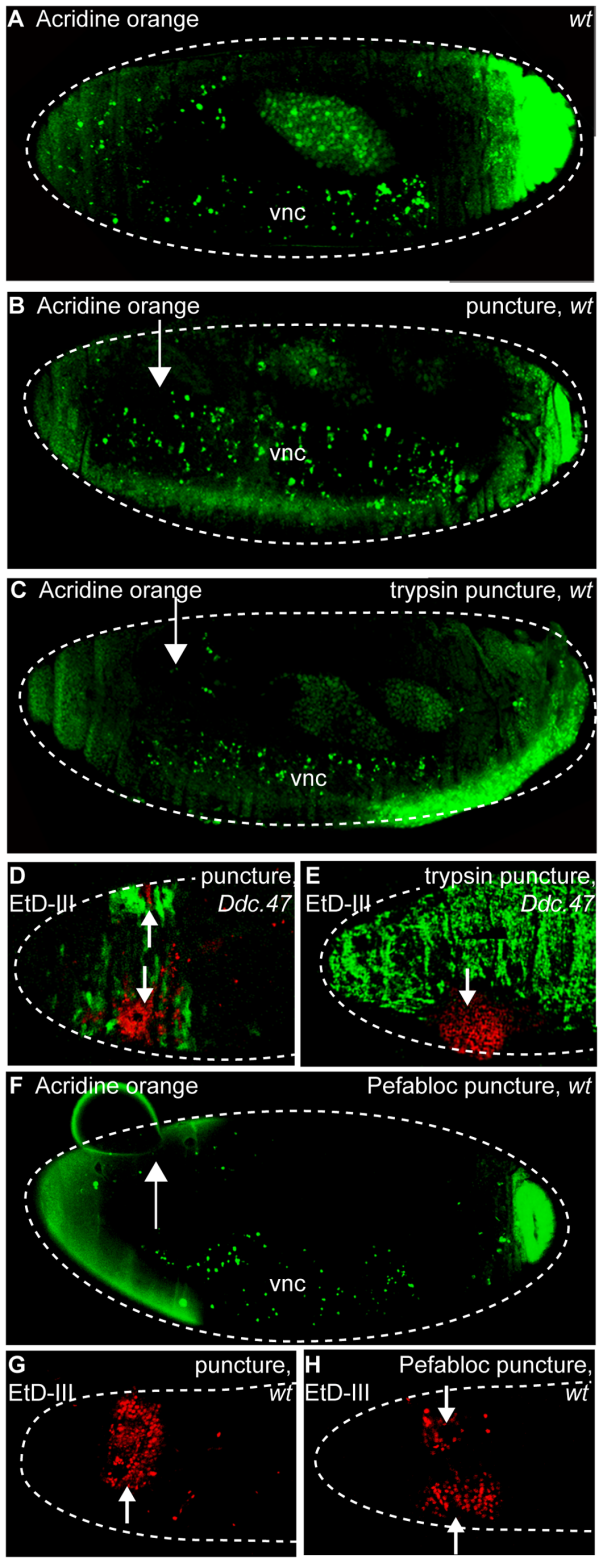
Trypsin or Pefabloc treatments do not cause widespread epidermal cell death. Confocal images of embryos stained with acridine orange (apoptosis marker) and Ethidium homodimer-III (EtD-III, necrosis marker) two to five hours after wounding. *Ddc*.47 is a fluorescent reporter that includes a wound-induced DNA enhancer from the *Ddc* locus. (A) Wild-type unwounded embryos exhibit normal acridine orange (green) staining in the ventral nerve cord and brain region. (B, C, F) Similar acridine orange staining is observed in puncture (both water and HCl trypsin buffer), trypsin puncture wounded, and Pefabloc puncture wounded embryos. Anterior and posterior pole staining is an artifact. (D) Puncture-only wounded *Ddc*.47 embryos activate reporter (green) around the wound site in the epidermis while EtD-III (red) stain is localized to the melanized scab. (E) Puncture-trypsin wounded embryos activate reporter globally throughout the epidermis, but EtD-III staining remains relatively localized to the puncture wound site. (G) Wild-type embryos puncture wounded with water exhibit EtD-III (red) stain localized to the wound site. (H) Pefabloc puncture wounded embryos exhibit a slight expansion of EtD-III staining around the wound site compared to puncture wounded without Pefabloc (G). vnc = ventral nerve cord. Arrows mark the wound site. Dashed white lines outline embryos.

To test whether trypsin treatment activates a global epidermal wound response by inflicting cell death, we stained trypsin-treated embryos with apoptosis and necrosis markers and compared them to wild-type controls and puncture-wounded controls without trypsin. Normal developmental apoptosis can be detected with acridine orange (AO) in the brain region and in the ventral nerve cord of stage 15 wild-type *Drosophila* embryos [33]. We could detect no changes in levels of apoptosis in puncture-trypsin treated embryos when compared to wild-type or puncture-only wounded controls at the same stage (Figure 4A–C). To test whether cellular necrosis levels were increased after puncture wounding with trypsin, embryos with wound reporters were stained with Ethidium homodimer-III (EtD-III) and compared with puncture-wounded controls without trypsin [34]. Puncture wounded embryos have localized necrosis at and near the melanized plug at wound sites (Figure 4D). Puncture-trypsin treated embryos had only a slightly expanded zone of necrosis around the puncture site (Figure 4E). Taken together, it appears that trypsin treatment is not activating a global epidermal wound response by inflicting widespread apoptosis or necrosis.

We also wished to test whether the serine protease inhibitor Pefabloc might be inhibiting wound reporter activation by triggering an expanded zone of epidermal cell death near puncture wounds. We could detect no epidermal apoptosis in Pefabloc treated wild-type embryos when compared to wild-type puncture wounded controls at the same stage during late embryogenesis (Figure 4F, B). In addition, Pefabloc treated embryos had a zone of necrosis around wound sites that was very similar to puncture-only control embryos (Figure 4G, H). We conclude that wounding with Pefabloc does not inhibit wound reporter activation by inflicting widespread apoptosis or necrosis.

### Serine Protease Activity is Required Both for the Initiation as Well as the Spatial Expansion of Epidermal Wound Response Transcription

**Figure 5:**
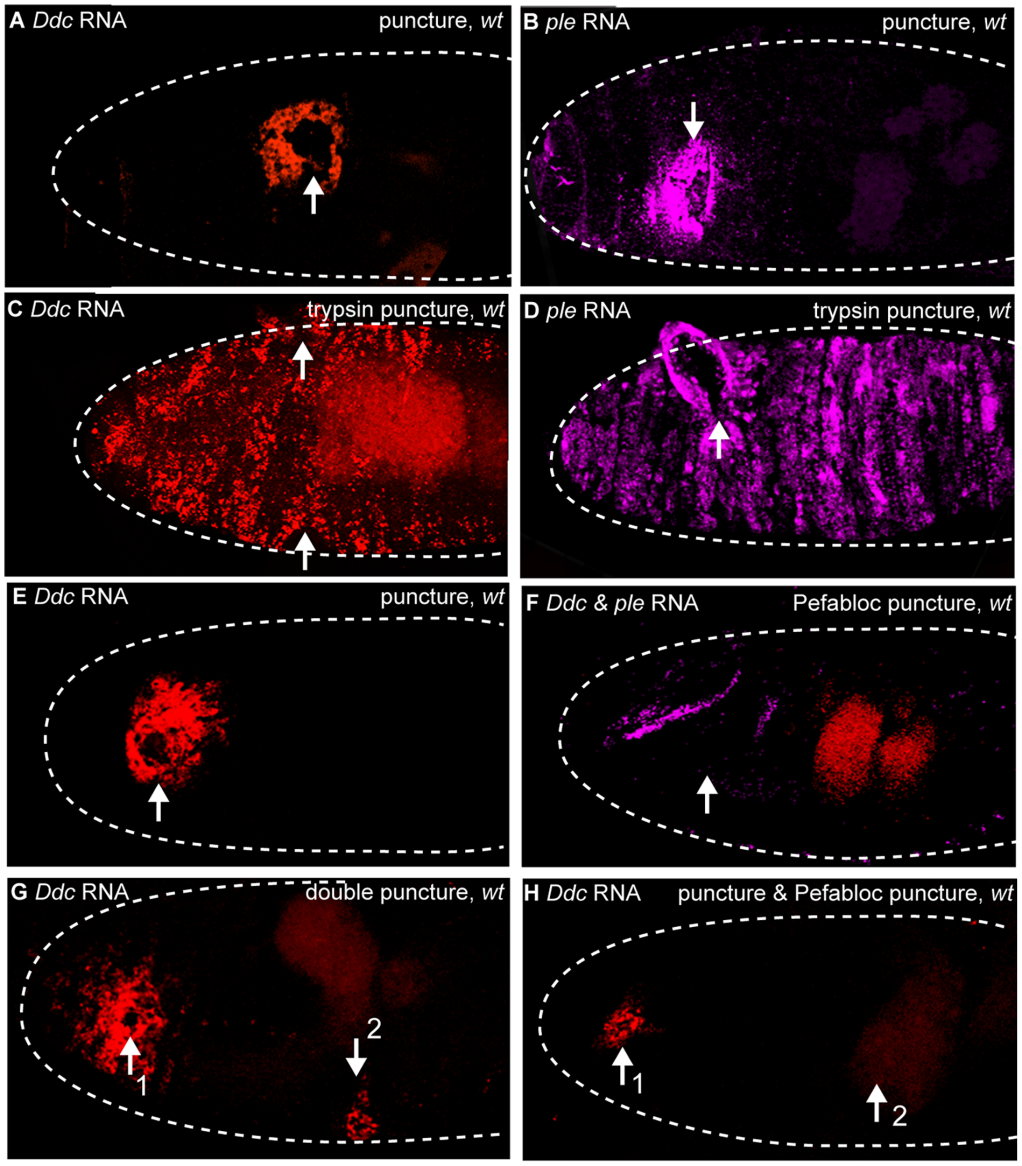
Serine proteases are sufficient and required for epidermal wound response gene transcription. Confocal images of wild-type embryos after *in situ* hybridization with fluorescently labeled RNA probes made to detect transcripts from *ple* (magenta) and *Ddc* (red). (A, B) 30 minutes after HCl (trypsin buffer) puncture wounding, *Ddc* and *ple* transcripts accumulate in the epidermis around the wound site. (C, D) 30 minutes after puncture-trypsin wounding, *ple* and *Ddc* transcript accumulation can be observed throughout a large region of the epidermis. (E) One hour after water puncture wounding, wild-type embryos activate *Ddc* transcripts in the epidermis surrounding the wound site. (F) One hour after Pefabloc puncture wounding, no *Ddc* transcripts are activated in the epidermis surrounding the wound site in wild-type embryos, but normal developmental expression of *ple* in the cells that secrete the head skeleton is observed (magenta). Gut autofluorescence is seen in red. (G) In wild-type, double water puncture wounded embryos, 60 minutes after the first water puncture wound, a moderately wide zone of *Ddc* transcripts in the epidermis around wound sites is observed, while 30 minutes after the second wound, a narrow zone of *Ddc* transcript accumulation is observed around the wound site. (H) In wild-type double puncture wounded embryos with Pefabloc injected at the second site, 60 minutes after the first puncture wound, a narrow zone of accumulation of *Ddc* transcripts in the localized epidermis is observed around the first wound site, while 30 minutes after the second puncture wound with Pefabloc, no *Ddc* transcript accumulation is observed at the wound site. Arrows mark the wound sites. “1″ and “2″ indicate first and second wounds, respectively. Dashed lines in the data panels mark the outlines of embryos.

The fluorescent proteins produced by our wound reporter transgene constructs are not detectable until 3–5 hours after puncture wounding. To test whether trypsin and Pefabloc treatments affected the initiation or propagation phases of wound gene activation, we conducted RNA *in situ* hybridization after trypsin or Pefabloc treatment. Thirty minutes after puncture wounding with trypsin, both *Ddc* and *ple* transcripts are upregulated in very broad zones of epidermal cells around wound sites, while embryos puncture wounded with buffer solution upregulate *ple* and *Ddc* transcripts in very localized zones (∼1–3 cell diameters) around wound sites (Figure 5A–D). As a control for probe trapping at wound sites, we did *in situ* hybridizations on transcript null mutants of *ple* and *Ddc* and saw no signal with the *in situ* probes for these genes at wound sites (unpublished data). In summary, 30 minutes of trypsin treatment (at the concentration we used, Materials and Methods) is sufficient to activate of *Ddc* and *ple* transcription during the initial phase of wound-induced transcription, in cells far beyond the normal epidermal zone observed after puncture-only wounding.

To test whether serine protease activity was required for the initial phase of wound-induced transcription, we performed RNA *in situ* hybridization with *ple* and *Ddc* probes on Pefabloc treated embryos. As expected, one hour after puncture wounding with carrier solution, a zone of epidermal cells ∼3–5 cell diameters around puncture sites activate *Ddc* or *ple* transcription (Figure 5E, unpublished data). No wound gene transcription is detected at wound sites one hour (or at 30 minutes, see below) after puncture wounding with Pefabloc (Figure 5F). As a positive control for *in situ* quality, the developmental expression of *ple* transcripts is detected in the head skeleton and anal pad in the Pefabloc treated embryos (Figure 5F, unpublished data). Thus, endogenous serine protease activity is required for the initiation phase of wound-induced *Ddc* transcription.

Although Pefabloc can inhibit the initial phase of wound induced transcription, we hypothesized it might also affect the spread of wound gene activation in epidermal cells. To test this, we performed RNA *in situ* hybridization for wound gene activation on individual embryos punctured at two time points. One hour after the first puncture wound, *Ddc* transcripts accumulate in a radial zone about 3–5 cells wide around wound sites, while a narrower zone of wound transcription is seen thirty minutes at the site of the second puncture wound within the same embryo (Figure 5G). We then performed an identical double wounding protocol, except that the second puncture wound was done with addition of Pefabloc (Figure 5H). As expected, at the Pefabloc wound site we did not observe any epidermal *Ddc* transcription. Remarkably, at the 1st wound site, where wound transcription had one hour to accumulate, but had at most thirty minutes to respond to the influence of Pefabloc, there was only a narrow zone of wound-dependent *Ddc* transcripts (compare wound transcription at wound site 1 in Figure 5G to wound site 1 in Figure 5H). These results indicate that serine protease activity is required for both the initiation and expansion of wound-dependent *Ddc* epidermal transcription.

### Placing Serine Protease Function in an Epidermal Wound Response Signaling Pathway

Hydrogen peroxide is one wound-induced signal that can attract blood cells to the site of clean epidermal wounds in zebrafish larvae and *Drosophila* embryos [22], [23]. The Duox enzyme and the hydrogen peroxide it produces are also part of the signaling pathway that leads to the activation of epidermal wound response genes in *Drosophila* embryos, and since trypsin can activate such genes even when Duox is absent, it has been proposed that hydrogen peroxide acts upstream of serine proteases in an epidermal wound response pathway [14].

**Figure 6:**
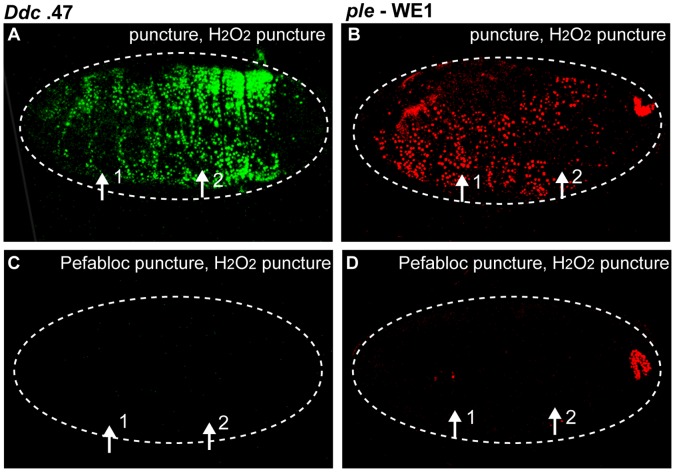
Serine protease activity is downstream of hydrogen peroxide with respect to wound reporter activation. Confocal images of *Ddc*.47 and *ple-*WE1 embryos that have been double puncture wounded with hydrogen peroxide and/or Pefabloc. (A, B) Embryos that have been water puncture wounded first and then wounded with hydrogen peroxide second exhibit global reporter activation. (C, D) Embryos that have been wounded first with Pefabloc and second with hydrogen peroxide do not activate reporter at either wound site. *Ple*-WE1 developmental anal pad expression is observed in each treatment. The numbers “1″ and “2″ indicate the first and second wound sites, respectively. Arrows mark the wound site(s). Dashed lines outline the embryos. *Ddc*.47 and *ple*-WE1 are fluorescent reporters that include wound-induced DNA enhancers from the *Ddc* and *ple* loci, respectively.

To further test this hypothesis, we performed double puncture wounding assays where we simultaneously blocked serine protease activity and introduced hydrogen peroxide within individual reporter embryos. Embryos that have been puncture wounded with carrier solution, then injected with hydrogen peroxide show global activation of wound reporters in the epidermis (Figure 6 A, B). However, embryos that have been first wounded with a needle filled with Pefabloc, then injected with hydrogen peroxide do not activate wound reporters (Figure 6 C, D). This provides additional evidence that hydrogen peroxide acts upstream of serine protease(s) in an epidermal wound gene activation pathway. Hydrogen peroxide can also activate epidermal wound reporters globally when applied only to the apical side of epidermal cells, indicating that the mere presence of hydrogen peroxide in the absence of puncture wounding is sufficient to induce a wound transcriptional response (Figure S3A, B). Taken together, we conclude that hydrogen peroxide or serine protease(s) can induce wound gene transcription without cellular breakage, and they act in series to promote epidermal wound gene activation.

**Figure 7:**
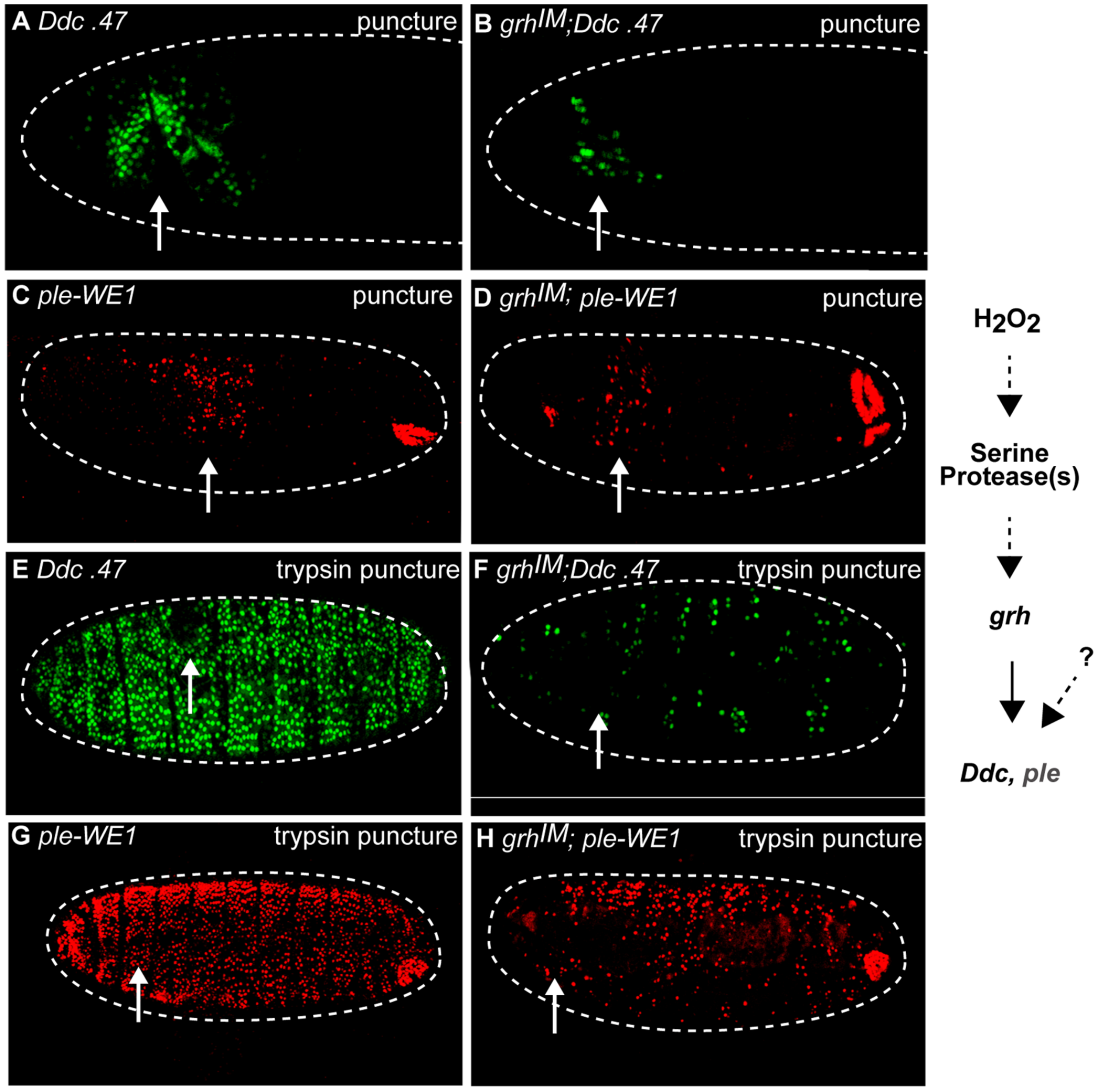
Serine protease-mediated wound reporter activation is upstream of *grainyhead* function. Confocal images of control *Ddc*.47 and *grh^IM^* mutants; *Ddc*.47 or *grh^IM^*; *ple* -WE1 activation about six hours after puncture and trypsin puncture wounding. *Ddc*.47 and *ple*-WE1 are fluorescent reporters that include wound-induced DNA enhancers from the *Ddc* and *ple* loci, respectively. (A, B) *Ddc*.47 embryos puncture wounded with carrier solution activate localized reporter at the wound site, but dramatically reduced localized reporter activation is observed in *grh* mutants after the same treatment. (C, D) *Ple-*WE1 embryos puncture wounded with carrier solution activate reporter around the wound site, while *grh* mutants exhibit only slightly reduced reporter activation at the wound site. The developmental anal pad expression from the *ple-*WE1 reporter construct is observed in each treatment. (E, F) Puncture-trypsin wounded *Ddc*.47 embryos activate reporter globally, while *grh* mutants exhibit dramatically reduced and scattered wound reporter activation after trypsin treatment. (G, H) Trypsin-treated *ple*-WE1 embryos activate reporter globally, while *grh* mutants activate lower, patchier, but still easily detectable global reporter activation after trypsin treatment. Developmental *ple* -WE1 anal pad expression is observed in every treatment. The pathway is shown on the right side of the figure. Arrows mark the wound site. Dashed lines in the data panels mark the outlines of embryos.

The Grh transcription factor is known to regulate the localized activation of a number of epidermal wound response genes in *Drosophila* embryos [6], [7], [14], [16], [19]. In order to test if serine protease activity was a component of the Grh-dependent epidermal wound response pathway, we compared *Ddc* and *ple* reporter activation levels between trypsin-treated *grh* null mutant and control embryos. Previous work has indicated that *Ddc* is more dependent on *grh* function than is *ple* for wound-dependent induction since the *ple* wound reporter can still be activated, although at lower levels, at the site of epidermal wounds in *grh* mutant embryos [6], [7]. As expected, after puncture-only wounding *grh* embryos with needles filled with carrier solution, we observed strikingly reduced *Ddc* wound reporter activation at wound sites, and moderately reduced *ple* wound reporter activation, compared to wounded wild-type controls (Figure 7A–D). In contrast, in puncture-trypsin wounded *grh* embryos, only weak, scattered *Ddc* wound reporter activation was observed, while wild-type controls showed robust, global wound reporter expression (Figure 7E, F). A modestly reduced number of epidermal cells activate the *ple* wound reporter in *grh* mutants after puncture-trypsin treatment, consistent with a weaker *grh* requirement for activation of the *ple* wound enhancer [6] (Figure 7G, H). The late embryonic anal pad expression pattern from the *ple* wound reporter transgene is observed in the *grh* mutant background indicating that *grh* mutants progress at similar developmental rates compared to control embryos (Figure 7G, H). Taken together, these results indicate that serine protease-induction of the *Ddc* and *ple* wound reporters is upstream of *grh* function.

### The Wound Transcriptome Utilizing Trypsin as a Global Epidermal Wounding Tool

Based on the above, our trypsin treatment protocol does not elicit a global wound response by breaching the epidermal barrier, or inflicting cellular damage or death. Furthermore, the requirement for a trypsin-like serine protease in an established wound response pathway downstream of *Duox* and hydrogen peroxide and upstream of *grh, Ddc*, and *ple* indicates that trypsin is most likely mimicking an endogenous signal for wound gene activation. Thus, we used trypsin treatment as an advantageous tool to globally wound the epidermis, and increase the efficiency of discovering the overall transcriptional response to epidermal wounds.

Microarray-based transcriptome profiles of puncture-only wounded and puncture-trypsin wounded stage 15–17 wild-type embryos were generated and compared to the transcriptome profiles of untreated wild-type stage 15–17 embryos. Three time points were analyzed, 30, 60, and 120 minutes after wounding. The 30 minute time point was chosen to analyze genes involved during the early stages of the wound healing process that are immediate targets of transcriptional activation. The 120 minute time point was chosen to determine genes involved during later stages of wound healing (epidermal wound closure is completed approximately two hours after puncture wounding) [35]. From the microarray absolute intensity values, false discovery rate (FDR) tests identified several hundred statistically significant (FDR<0.01) differentially expressed genes for each treatment and time point in relation to control wild-type embryos. Based on scatter plot analysis, the microarray data was found to be highly reproducible between biological replicate samples for both puncture-only and puncture-trypsin wounding treatments (Figure S4A, B).

### Transcriptome Changes after Puncture and Trypsin Puncture Wounding Treatments

**Figure 8:**
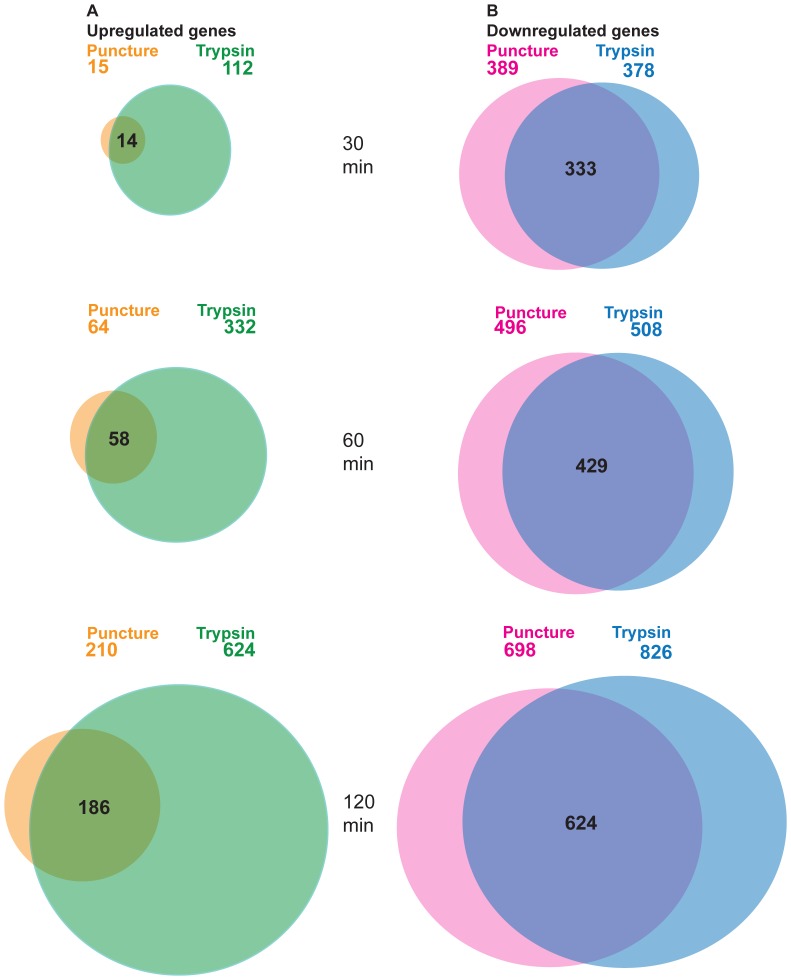
Abundant overlap of differentially regulated genes after puncture and trypsin puncture wounding. At each of the three time points (30 minutes, 60 minutes, and 120 minutes) after puncture and trypsin puncture wounding, a comparison of the statistically significant (FDR <0.01) regulated genes was performed using Microsoft Excel software. (A) 30 minutes after puncture and trypsin puncture wounding, 15 and 112 significant genes respectively, were upregulated 1.8 fold or greater; 14 genes were commonly upregulated after both wounding treatments. 60 minutes post puncture and trypsin puncture wounding, 64 and 332 significant genes respectively, were upregulated 1.8 fold or greater; 58 genes were commonly upregulated after both wounding treatments. 120 minutes post puncture and trypsin puncture wounding, 210 and 624 significant genes respectively, were upregulated 1.8 fold or greater; 186 genes were commonly upregulated after both wounding treatments. (B) 30 minutes post puncture and trypsin puncture wounding, 389 and 378 significant genes respectively, were downregulated −1.8 fold or lower; 333 genes were commonly down regulated after both wounding treatments. 60 minutes post puncture and trypsin puncture wounding, 496 and 508 significant genes respectively, were downregulated −1.8 fold or lower; 429 genes were commonly downregulated after both wounding treatments. 120 minutes post puncture and trypsin puncture wounding, 698 and 826 significant genes respectively, were downregulated −1.8 fold or lower; 624 genes were commonly downregulated after both wounding treatments.

Assayed during late embryogenesis, puncture-only and puncture-trypsin wounding had a large impact on the *Drosophila* transcriptome. Transcripts that were upregulated more than 1.8 fold, and had a FDR<0.001, we arbitrarily classified as highly significant. The Venn diagrams in Figure 8A visually display the amazing similarity in the upregulated genes when comparing puncture and puncture wounding with trypsin. At every time point, between 88–93% of genes upregulated by puncture wounding were also upregulated in response to trypsin puncture wounding. Most importantly, of the genes upregulated by puncture-only wounding, a very high percentage (81% at 120 minutes after wounding) showed an increased fold change after trypsin puncture wounding (Table S2). For downregulated genes, transcripts that were downregulated more than 1.8 fold, and had a FDR<0.01 were classed as significant. At every time point, between 85–89% of the genes downregulated by puncture-only wounding were also downregulated in response to trypsin puncture wounding (Figure 8B). These comparisons suggest that trypsin wounding robustly amplifies the puncture-only wounding transcriptional profile, and suggests that trypsin wounding would allow detection of additional genes that are locally activated around epidermal puncture wounds. Puncture wounding plus trypsin is also likely to induce the transcriptional activation of a greater number of genes in part because of trypsin’s ability to globally wound different internal tissues and activate wound induced genes in one or more of those tissues.

### Enriched Gene Ontology Categories for the Regulated Genes from the Puncture and Trypsin Puncture *Drosophila* Embryo Microarrays

A search for enriched Gene Ontology (GO) “Biological Process” and “Molecular Function” categories was performed (Text S1). At the 120 minute time point, the 17 most significant classes of enriched GO terms associated with genes significantly upregulated after puncture and trypsin puncture wounding included cuticle repair, epidermal re-epithelialization, and melanization processes (Table 1, unpublished data). That these biological categories were over-represented indicates that our wounding protocol is capturing genes involved in epidermal regeneration. Many GO terms related to general defense responses, including innate immunity signaling pathways, were also highly over-represented in both the upregulated puncture and trypsin puncture wounding gene lists (Table 1). Another type of GO term related to general defense response that was significantly enriched in upregulated puncture and trypsin puncture wounding profiles was a stress response, presumably as a result of tissue damage (Table 1). Other significantly enriched GO terms associated with upregulated puncture and trypsin puncture gene sets included genes encoding either serine proteases or serine protease inhibitors. Serine protease cascades trigger melanization reactions in response to infection, and serine protease inhibitors (serpins) are used to restrict melanin deposition (for a review see [36]).

**Table 1:** Enriched Gene Ontology terms for regulated genes from the *Drosophila* microarrays.

Upregulated GO term	GO term ID	# genes	p-value
defense response	6952*	45	1.85E-22
response to other organism	51707*	38	3.15E-20
response to bacterium	9617*	27	4.95E-18
immune response	6955*	38	1.01E-17
serine-type endopeptidase activity	4252*	48	6.21E-15
serine-type endopeptidase inhibitor activity	4867*	22	9.24E-14
response to stress	6950	67	9.81E-14
response to fungus	9620	13	2.64E-10
regulation of immune response	50776*	12	5.33E-08
aminoglycan metabolic process	6022	22	6.90E-08
Toll signaling pathway	8063*	11	1.21E-07
regulation of Toll signaling pathway	8592	7	5.49E-07
carbohydrate binding	30246	24	5.95E-07
carbohydrate metabolic process	5975	40	1.09E-06
hydrolase activity	16787*	130	3.85E-06
lipase activity	16298*	16	5.91E-06
glutathione transferase activity	4364	10	7.84E-06
**Downregulated GO term**	**GO term ID**	**# genes**	**p-value**
chromosome organization	51276*	75	3.11E-34
organelle organization	6996*	132	2.11E-27
cellular component organization	16043*	188	6.54E-26
nucleic acid binding	3676*	178	1.18E-24
cell cycle	7049*	86	5.63E-23
cellular component biogenesis	44085	75	2.41E-18
cellular biopolymer metabolic process	34960*	177	1.81E-17
nucleotide and nucleic acid metabolic process	6139*	111	1.33E-16
chromatin modification	16568	28	8.51E-16
regulation of cell cycle	51726*	37	2.29E-14
anatomical structure formation	10926*	81	3.13E-14
RNA metabolic process	16070*	68	7.20E-14
chromosome segregation	7059*	29	1.44E-13
negative regulation of biological process	48519*	70	8.18E-13
oogenesis	48477*	58	1.13E-12
regulation of macromolecule metabolic process	60255*	108	1.36E-12

Enriched Gene Ontology (GO) categories for the 624 significantly upregulated and 826 significantly downregulated (FDR<0.01) genes from *Drosophila* Agilent microarrays at the 120 minute time point after trypsin puncture wounding. Each GO term is associated with a GO term ID. The number of genes regulated per GO term is listed, as is the corresponding p-value for statistical significance measurement (p<0.05).

* Denotes that identical GO term ID was enriched (p<0.05) at 120 minutes after puncture wounding.

The 16 most significant classes of enriched GO terms associated with genes significantly downregulated 120 minutes after puncture-only and puncture-trypsin wounding are also shown in Table 1. The majority of these enriched GO terms include genes that regulate chromosome structure, the cell cycle and developmental patterning (Table 1). From these results we propose that after injury via puncture or trypsin puncture wounding, embryonic development is briefly delayed, in part by inhibition of DNA replication and growth, so that embryos can perform the repair of small wounds, and mobilize a response to fight pathogens that enter through wound sites.

### Upregulated Genes after Puncture Wounding and Trypsin Puncture Wounding of Late-stage *Drosophila* Embryos

We carried out a manual classification for 84 genes upregulated 30 minutes, 60 minutes and/or 120 minutes after puncture and/or trypsin puncture wounding on the *Drosophila* embryo microarrays. These 84 genes were selected because they had very significant fold change values, and had biological functions that could be rationalized as being related to wound repair, for example, the processes of re-epithelialization, cell adhesion, cuticle repair, and defense against microbial infection (in the wild, microbial entry always accompanies puncture wounding). In Table 2 we show these grouped in the categories of: “Cuticle Regeneration/Chitin Metabolism, Melanization, Innate Immunity, Epidermal Wound Response, Cytoskeleton/Cell Adhesion, Detoxification/Defense/Stress Response, Serine Proteases & Serpins, and Signaling/Miscellaneous”. Verification of microarray fold change directionality for 11 genes using quantitative RT-PCR is shown in Figure S5.

**Table 2:** Select upregulated genes from the trypsin puncture wound microarrays.

CG #	Gene symbol	Protein type/Process	30 min Fold change	60 min Fold change	120 min Fold change	CG #	Gene symbol	Protein type/Process	30 min Fold change	60 min Fold change	120 min Fold change
Cuticle Regeneration/Chitin metabolism (10/19)						Epidermal Wound Response (7/10)					
CG13224	Cpr47Eb	cuticle protein	4.2	18.3*	29.8*	CG11086	Gadd45	damage inducible protein	15.3*	7.6*	6.5*
CG9077	Cpr47Ec	cuticle protein	2.0	11.9*	23.3*	CG2914	Ets21C	ETS transcription factor	1.9	5.4*	4.9*
CG7539	Edg91	cuticle protein	2.8	7.4	12.3*	CG33956	kay	FOS transcription factor	2.6	4.5*	2.9
CG10118	ple	cuticle regeneration	5.6*	12.9*	9.5*	CG2275	jra	JUN transcription factor	1.5	2.4	2.3*
CG1864	Hr38	cuticle development	N/A	6.6*	8.1*	CG7873	Src42A	protein tyrosine kinase	N/A	1.7	2.1
CG9076	Cpr47Ed	cuticle protein	N/A	3.0	8.1	CG7850	puc	JNK pathway phosphatase	1.4	2.1	1.7
CG10697	Ddc	cuticle regeneration	2.0	4.3*	5.3*	CG10244	Cad96Ca	Stitcher RET RTK	N/A	1.8	N/A
CG2666	kkv	chitin synthase	N/A	2.3	3.1	**Cytoskeleton/Cell adhesion (6/12)**					
CG33983	obst-H	chitin binding protein	N/A	N/A	2.3	CG6449	NijA	cell adhesion	N/A	2.5	4.1*
CG15378	lectin-22C	chitin metabolism	N/A	N/A	1.8	CG8095	scb	cell adhesion molecule	1.5	3.4	3.8
**Melanization (5/5)**						CG5730	AnnIX	actin binding	2.1*	2.3	2.7*
CG9733	CG9733	protease, proAE	6.0	14.1*	18.9*	CG12051	Act42A	actin	N/A	N/A	2.1
CG1689	lz	crystal cell differentiation	N/A	8.7*	14.0*	CG4755	RhoGAP92B	Rho GTPase activator	1.4	N/A	1.7
CG1102	MP1	protease/activator	1.7	2.3	3.7	CG4027	Act5C	actin	N/A	N/A	1.7
CG3066	Sp7	protease/activator	N/A	1.7	3.4*	**Detoxification/Defense/Stress response (8/30)**					
CG11331	Spn27A	serpin	N/A	1.7	2.1	CG4421	GstD8	glutathione S transferase	1.7	7.5	12.8
**Innate immunity (29/31)**						CG3666	Tsf3	iron sequestration	4.8*	12.2*	9.8*
CG18372	AttB	AMP (bacterial)	43.7*	120.1*	73.6*	CG4371	GstD7	glutathione S transferase	N/A	3.3	7.5*
CG15066	IM23	putative AMP	21.5*	83.6*	56.2*	CG10363	TepIV	humoral response	1.7	3.4*	4.2*
CG18106	IM2	putative AMP	32.5*	76.1*	49.1*	CG7052	TepII	opsonization	N/A	2.1	3.2
CG4740	AttC	AMP (bacterial)	3.4	10.7*	44.1*	CG8913	Irc	Immune regulated catalase	1.6	1.9	3.0*
CG10146	AttA	AMP (bacterial)	11.6	50.2*	40.3*	CG6965	mthl5	GPCR	1.6	3.5*	2.5
CG10810	Drs	AMP (fungi)	5.8*	27.2*	39.0*	CG4026	IP3K1	oxidative stress response	2.6	1.7*	2.1*
CG18108	IM1	putative AMP	21.4*	57.2*	38.9*	**Signaling/Miscellaneous (12/25)**					
CG16844	IM3	putative AMP	19.9*	48.7*	27.4*	CG1851	Ady43A	adenosine kinase activity	15.3*	40.2*	9.2*
CG15231	IM4	putative AMP	20.7*	41.9*	28.0*	CG33542	upd3	JAK-STAT signaling	N/A	5.6*	4.6*
CG8175	Mtk	AMP (fungi)	5.1	12.4*	25.9*	CG5993	os	JAK-STAT signaling	2.7	5.8*	4.2*
CG18279	IM10	putative AMP	11.2*	27.6*	18.4*	CG9811	Rgk1	GTPase-mediated signaling	1.9*	1.8*	3.6*
CG10794	DptB	AMP (GP bacteria)	5.0	6.6*	7.5*	CG33338	p38c	MAP kinase	1.6	2.3	3.4
CG11992	Rel	NFkB transcription factor	3.4	7.9*	6.4*	CG7450	CrebA	DNA binding	2.5*	2.7*	2.6*
CG11709	PGRP-SA	PRR	1.5	2.7	6.2*	CG5248	loco	GPCR signaling	1.4*	2.1*	2.3*
CG1857	nec	serpin/Toll signaling	2.4	3.8*	6.1*	CG6117	Pka-C3	protein kinase	N/A	N/A	2.3
CG4437	PGRP-LF	PRR	7.3*	10.7*	5.5*	CG1147	NPFR1	Neuropeptide Y receptor	N/A	N/A	2.2
CG6134	spz	Toll signaling ligand	1.8	4.6	5.5*	CG6103	CrebB-17A	DNA binding	1.4	1.6	2.2
CG16705	SPE	protease/Toll signaling	1.7	2.9*	4.0*	CG4472	Idgf1	imaginal disc development	1.4	2.3	2.2*
CG2056	spirit	protease/Toll signaling	1.5	2.2	3.5*	CG1004	rho	EGFR pathway activator	N/A	1.8	N/A
CG14704	PGRP-LB	PRR	2.0	4.7*	3.4*	**Serine proteases & Serpins (7/16)**					
CG32042	PGRP-LA	PRR	1.3	1.9	2.8*	CG33329	Sp212	serine protease	1.5	3.6	7.6*
CG5848	cact	protein binding/Toll signaling	1.4	2.0	2.5	CG2045	Ser7	serine protease	N/A	4.1*	6.8*
CG1165	LysS	lysozyme activity	N/A	N/A	2.6	CG18525	Spn5	serpin	1.5	2.7	4.9*
CG1365	CecA1	AMP (bacterial)	N/A	1.7*	2.5*	CG4821	Tequila	Neurotrypsin ortholog	N/A	N/A	3.9*
CG4432	PGRP-LC	PRR	2.5*	2.6*	2.4*	CG10913	Spn6	serpin	N/A	2.1	3.3
CG6367	psh	protease/Toll signaling	1.5	1.8*	2.4*	CG7996	snk	protease/Toll signaling	N/A	1.6	2.4*
CG6667	dl	NFkB transcripton factor	N/A	2.7	2.1*	CG9453	Spn4	serpin	1.4	2.1	2.9*
CG1373	CecC	AMP (bacterial)	N/A	1.8*	2.1*						
CG6794	Dif	NFkB transcripton factor	N/A	1.6	N/A						

A total of 84 significantly upregulated genes after trypsin puncture wounding were manually classified into the above labeled categories. The categorized genes are based on 120 minute significant fold change values since this timepoint contained the highest amount of upregulated genes after either wounding treatment. Category headings denote the number of genes manually listed in the table out of the total number of upregulated genes 120 minutes after trypsin treatment that fall into the same category heading. “CG #” refers to the accession numbers from Flybase. “Gene symbol” refers to gene symbol on Flybase. “Protein type/Process” refers to experimentally verified or putative functions assigned to genes. “Fold change” refers to fold changes seen in gene expression values (either puncture or trypsin puncture treatments) relative to wild-type untreated values. All genes shown are statistically significant and have a FDR value of less than 0.01. PRR, Peptidoglycan Recognition Receptor. N/A indicates that a statistically significant fold change was not achieved for the corresponding gene at that specific timepoint or that duplicate probe values were not reproducible. *Denotes gene that is significantly upregulated at corresponding timepoint after puncture wounding with a FDR less than 0.01.

Table 2 shows 10 of the genes in the cuticle regeneration category that are upregulated in response to puncture and/or puncture-trypsin wounding treatments. These genes are involved in chitin metabolism, and the production of cuticle proteins [37]. At the 120 minute time point the highest fold upregulation is seen for almost all 10 genes, suggesting that genes involved in cuticle repair and metabolism are largely late wound response genes (Table 2, unpublished data). *Lectin-22C* is a significantly upregulated gene after trypsin wounding; lectins are classes of sugar recognition molecules that mediate cellular and cell–substrate interactions [38]. They also confer signals to the immune system which allow an organism to distinguish self determinants from non-self or modified-self determinants [39]. There is evidence that *Drosophila* lectins can be substrates for transglutaminase crosslinking enzymes and play a role in cuticle morphogenesis [40]. Perhaps after clean puncture wounding induces cuticular damage, *lectin-22C* is upregulated for dual roles in cuticle repair, as well as for self-recognition during the response to infectious wounds. *Kkv* is a previously identified localized epidermal wound response gene that encodes chitin synthase [6], and was significantly upregulated at two time points after puncture-trypsin wounding, but was not detected as significant after puncture-only wounding, further validating our use of trypsin as a useful tool to identify genes activated in a few epidermal cells after puncture-only wounds.

Also consistent with a wound phenotype, five genes known to be involved in a category we called “Melanization” were significantly upregulated at one or more time points after puncture or trypsin puncture wounding (Table 2). This category includes 3 serine proteases, including *MP1* and *Sp7* (also known as *MP2*), which are two immune inducible serine proteases which act in a melanization cascade along with the serpin *Spn27A* to encapsulate and kill potential microbial pathogens that may enter the host wound site [24]. Also included in this category is CG9733, which encodes a prophenoloxidase activating enzyme, a serine protease that activates phenoloxidase, a key enzyme in the melanization pathway [41].

The most spectacular fold change values spanning all 3 time points are contained in the category “Innate Immunity”: 29 of these genes are listed in Table 2. Both clean puncture and trypsin puncture wounding activate massive innate immune responses, as 14 known or putative antimicrobial peptides, such as *AttB*, were among the most highly induced wound genes. The battery of genes upregulated after clean puncture wounding of embryos closely parallels the transcriptional activation changes seen in *Drosophila* adults that are exposed to septic injury [42]–[44]. Specifically, 22 out of the 30 “Innate Immunity” upregulated genes in Table 2 are also significantly induced after adult septic injury and/or fungal infection [42]. Most of the innate immune genes that we found to be activated by trypsin puncture wounding are significantly activated at the earliest time point and remain strongly induced for the remaining two time points, peaking in fold change at the 60 minute time point. Taken together, these results, like those of others [42]–[44], indicate that clean wounding is a powerful inducer of an innate immune transcriptional response. This is also supported by our observation that numerous genes in the category “Serine proteases and Serpins”, such as *Ser7* and *Spn5* were induced by trypsin wounding in embryos (Table 2), as well as by septic wounding of adults [42]. Most of these genes have unknown biological functions; some, like the gene encoding the *SPE* protease, are involved in regulation of innate immunity [25]. In terms of temporal profiles of wound-induced transcription, this category of genes appears to be highly variable, suggesting that different serine proteases and serpins function at different stages of wound repair (Table 2).

We highlight 7 genes in the category “Epidermal Wound Response”, which includes genes involved in epidermal re-epithelialization [45] (Table 2). Several of these genes have been previously established as localized epidermal wound response genes (*Gadd45, Src42A, Cad96Ca*), so the fact that many of them were significantly upregulated after puncture and/or trypsin puncture wounding gave us even more confidence in the validity of using our microarray data to identify genes locally induced in the epidermis as a response to clean wounding [6], [7], [14]–[16]. Most of the genes in the “Epidermal Wound Response” category were significantly induced at 30 minutes and peaked at 60 minutes after wounding. Previously identified epidermal wound response genes, like *flo-2* and *msn*, were not detected to be significantly upregulated after puncture or trypsin puncture wounding [6], [14]. This is likely a limitation of sampling transcriptome changes in experiments that involve RNA isolated from wounded whole embryos; constitutive expression of these genes in most or all cells, even in the unwounded state, is presumably preventing the detection of the higher levels of transcription that occur in a relatively small number of embryonic cells after wounding.

Six upregulated genes are in the category “Cytoskeleton/Cell Adhesion” including genes potentially involved in actin-based wound closure processes such as *Act42A, Act5C*, and *RhoGAP92B,* which are all expressed at moderate to extremely high expression levels during late embryogenesis (Table 2) (www.flybase.org). Dorsal closure and embryonic wound closure depend on actin cable formation and contraction [35], [46], [47]. The small GTPase *RhoA* functions during actin-based wound closure by causing disassembly of the actin cable to promote contractility and uniform epithelial cell advancement movements to close the wound gap [35], and *RhoGAP92B* might be involved in activating *RhoA* functions during re-epithelialization.

The “Signaling/Miscellaneous” category contains 12 genes known or potentially involved in various signaling pathways that likely influence wound healing processes (Table 2). One example is *rhomboid (rho)* which encodes a transmembrane serine protease that promotes the intramembrane cleavage of Spitz, a *Drosophila* EGFR ligand [48]. We suggest that *rhomboid* is transcriptionally upregulated at the localized epidermal wound site to activate EGFR-mediated re-epithelialization of the wounded epidermis [21].

Eight genes are highlighted in the “Detoxification/Defense/Stress Response” category, including genes involved in restoring homeostasis after an external assault triggers a general stress response (Table 2). For example, Glutathione-S-transferases (GSTs), like *GstD8* and *GstD7*, encode a family of multi-functional enzymes involved in the detoxification of endogenous compounds [49]. GSTs also play a role in oxidative stress, a condition cells experience when there is an increase in reactive oxygen species (ROS), which can be mimicked by exogenous application of superoxides and hydrogen peroxide [50]. In response to dietary hydrogen peroxide, third instar *Drosophila* larvae induced the midgut-specific activation of several GST genes, which probably ameliorate the effects of oxidative stress [51]. The “Detoxification/Defense/Stress Response” genes seen in Table 2 are largely activated 60 and 120 minutes after wounding.

### Downregulated Genes after Puncture Wounding and Trypsin Puncture Wounding of Late-stage *Drosophila* Embryos

We carried out a manual classification of a total of 78 genes that were downregulated 30, 60 and/or 120 minutes after puncture and/or trypsin puncture wounding in the *Drosophila* embryo microarrays. These genes were selected based on their biological and biochemical functions and on fold change values. In Table 3 we assign these genes to several categories including: “Cell Cycle/Cell Division, Oogenesis/Development, Chromosome Organization, and Signaling/Miscellaneous”. Verification of microarray fold change directionality for 2 of these genes using RNA *in situ* hybridization is shown in Figure S6. The 16 genes in the functional category “Chromosome Organization” included genes involved in nucleosome processes, facilitation of DNA replication, and general chromosome topology and structure. For example, *spn-E*, a gene with helicase activity that functions to separate strands of the DNA double helix during replication events, was significantly downregulated after puncture and trypsin puncture wounding [52].

**Table 3:** Select downregulated genes from the trypsin puncture wound microarrays.

CG #	Gene symbol	Protein type/Process	30 min Fold change	60 min Fold change	120 min Fold change	CG #	Gene symbol	Protein type/Process	30 min Fold change	60 min Fold change	120 min Fold change
Cell Cycle/Cell Division (19/52)						Chromosome Organization (16/48)					
CG3510	CycB	cell cycle process	*−8.2	*−29.4	*−21.0	CG13399	Chrac-14	response to DNA damage	*−5.4	*−5.1	*−8.0
CG5814	CycB3	cell cycle process	*−7.1	*−20.3	*−20.0	CG3068	aur	mitotic spindle organization	*−3.1	*−4.4	*−6.6
CG4454	Borr	mitotic cell cycle	*−6.6	*−14.1	*−17.1	CG4236	Caf1	nucleosome binding	−2.8	−4.3	−6.3
CG5940	CycA	cell cycle process	*−4.0	*−6.7	*−9.3	CG2207	Df31	histone binding	*−2.3	*−2.6	*−4.2
CG8171	dup	sister chromatid separation	*−3.5	*−6.1	*−8.3	CG6146	Top1	DNA topological change	*−2.0	*−2.5	*−4.0
CG5363	cdc2	cyclin-dependent kinase	*−3.1	*−4.3	*−6.6	CG12165	Incenp	centromere protein	*−2.2	*−3.2	*−3.9
CG8068	Su(var)2–10	G2/M transition checkpoint	*−3.2	*−4.3	*−6.0	CG7269	Hel25E	DNA/RNA helicase	*−1.7	*−2.0	*−3.3
CG9096	CycD	cell cycle process	*−2.5	*−2.7	*−5.2	CG7055	dalao	chromatin remodeling	*−1.7	*−2.0	*−3.0
CG7838	Bub1	spindle assembly checkpoint	*−3.0	*−3.5	*−4.4	CG5499	His2Av	DNA binding; Histone H2A	*−1.6	*−1.8	−2.9
CG7581	Bub3	spindle assembly checkpoint	−2.0	*−2.6	*−3.9	CG3158	spn-E	helicase activity	*−2.4	*−2.3	*−2.7
CG6759	cdc16	metaphase/anaphase	*−1.9	*−2.5	*−3.8	CG6875	asp	microtubule binding	*−1.8	*−2.2	−2.7
CG3938	CycE	cell cycle process	*−1.7	*−2.6	*−3.4	CG15319	nej	histone acetyltransferase	*−1.9	*−2.2	*−2.3
CG1258	pav	cell cycle process	*−1.7	*−2.1	*−2.6	CG33804	His1:CG33804	chromatin (dis)assembly	*−1.5	N/A	*−2.2
CG10712	Chro	spindle assembly checkpoint	−1.6	−1.7	*−2.6	CG18013	Psf2	DNA helicase activity	N/A	*−1.8	*−2.1
CG5083	Rbf2	regulation of S phase	*−1.6	*−1.7	*−2.5	CG18608	prod	chromosome condensation	N/A	N/A	*−2.0
CG17437	wds	G2/M transition checkpoint	N/A	*−2.0	*−2.3	CG6384	Cp190	chromatin insulator binding	−1.6	N/A	−2.0
CG4654	dp	DNA damage response	N/A	*−1.7	*−2.0	**Signaling/Miscellaneous (23/65)**					
CG9750	rept	G2/M transition checkpoint	N/A	N/A	−2.1	CG1389	tor	RTK	*−6.3	*−12.3	*−14.6
CG4824	BicC	microtubule organization	N/A	*−8.3	*−2.6	CG3227	insv	Notch signaling	*−5.1	*−8.7	*−11.2
**Oogenesis/Development (20/47)**						CG13345	tum	Wnt signaling	*−4.2	*−7.5	*−8.4
CG5052	pim	hindgut morphogenesis	*−8.1	*−22.4	*−17.1	CG6391	Aps	nucleotide metabolism	*−2.0	*−2.4	*−3.5
CG1034	bcd	oogenesis	*−8.0	*−20.6	*−16.8	CG8384	gro	transcription corepressor	*−2.1	*−2.3	*−3.5
CG12306	polo	reproductive cell process	*−6.3	*−14.3	*−15.6	CG9755	pum	EGFR signaling	*−1.7	*−1.6	*−3.0
CG4965	twe	embryo development	*−7.1	*−14.0	*−13.6	CG5452	dnk	nucleotide phosphorylation	*−1.7	*−2.1	*−3.0
CG9183	plu	egg activation	*−3.7	*−5.3	*−6.5	CG6137	aub	metabolic process	*−2.0	*−1.9	*−2.8
CG4711	squ	oogenesis	*−3.3	*−4.7	*−6.2	CG3619	Dl	Notch binding	*−1.6	*−2.1	*−2.7
CG10901	osk	germ cell development	*−2.1	*−4.5	*−4.3	CG18211	betaTry	serine type endopeptidase	*−6.1	*−5.9	−2.8
CG15010	ago	regulation of growth	*−2.4	*−2.9	*−3.6	CG11228	hpo	apoptotic process	*−1.7	*−1.8	*−2.5
CG10528	fs(2)ltoPP43	chorion eggshell formation	*−2.2	*−2.5	*−3.6	CG9556	alien	transcription corepressor	N/A	*−1.7	*−2.2
CG1372	yl	vitellogenesis; oogenesis	*−1.9	*−4.8	*−3.4	CG16785	fz3	Wnt-protein binding	N/A	N/A	−2.2
CG11518	pygo	pattern specification	*−2.0	*−2.3	*−3.1	CG7524	Src64B	protein tyrosine kinase	N/A	*−2.1	*−2.1
CG13076	Notum	wing disc pattern formation	N/A	−3.1	−3.1	CG12351	deltaTry	serine type endopeptidase	*−10.5	*−10.9	*−3.1
CG2534	cno	epidermis morphogenesis	*−1.8	*−2.1	−2.9	CG8573	su(Hw)	regulation of transcription	N/A	N/A	*−2.1
CG4799	Pen	lymph gland development	*−1.6	*−2.2	*−2.7	CG1072	Awh	nucleic acid binding	N/A	−1.7	−2.1
CG18582	mbt	photoreceptor development	*−1.9	*−2.2	*−2.7	CG9786	hb	transcription factor	*−1.6	*−1.9	*−2.1
CG15119	mip40	oogenesis	−1.7	*−2.1	*−2.6	CG11561	smo	smoothened signaling	N/A	−1.7	−2.1
CG10125	zpg	germ cell development	*−1.6	*−1.8	*−2.6	CG1487	krz	Notch signaling	*−1.5	*−1.6	*−1.9
CG11375	polybromo	eggshell chorion assembly	N/A	*−2.1	*−2.1	CG6210	wls	Wnt signaling	−1.5	N/A	−1.9
CG5786	ppan	imaginal disc development	−1.9	N/A	−2.1	CG3497	Su(H)	Notch signaling	*−1.5	N/A	*−1.9
CG18361	dsh	reproductive cell process	N/A	−1.7	*−2.0	CG7926	Axn	Wnt signaling	N/A	N/A	*−1.8

A total of 78 significantly downregulated genes after trypsin puncture wounding were manually classified into the above labeled categories. The categorized genes are sorted based on 120 minute significant fold change values since this timepoint contained the highest amount of upregulated genes after either wounding treatment. Category headings denote the number of genes manually listed in the table out of the total number of upregulated genes 120 minutes after trypsin treatment that fall into the same category heading. “CG #” refers to the accession numbers from Flybase. “Gene symbol” refers to gene symbol on Flybase. “Protein type/Process” refers to experimentally verified or putative functions assigned to genes. “Fold change” refers to fold changes seen in gene expression values (either puncture or trypsin puncture) relative to wild-type unwounded values. All genes shown have a FDR value less than 0.01. N/A indicates that a statistically significant fold change was not achieved for the corresponding gene at that specific timepoint. *Denotes gene that is significantly downregulated at corresponding timepoint after puncture wounding with a FDR less than 0.01.

Nineteen downregulated genes were associated with “Cell Cycle/Cell Division” functions (Table 3). The genes for several cyclins, which allow cells to progress through checkpoints at various stages of the cell cycle, were significantly downregulated following puncture and trypsin puncture wounding. The results are consistent with previous studies of wounded *Drosophila* embryos that found no activation of cell division in nearby epidermal cells [35].

The category “Oogenesis/Development” contains 20 genes involved in morphogenetic and reproduction processes (Table 3). For example, *ppan* was a gene significantly downregulated 30 and 120 minutes after trypsin puncture wounding. At the cellular level, *ppan* is not absolutely required for growth or mitosis, but its absence does confer a growth delay, and it is also required for some aspects of normal cell differentiation and DNA replication in *Drosophila* larvae [53].

Twenty-three genes with transcriptional profiles that significantly decreased after wounding were associated with the “Signaling/Miscellaneous” category (Table 3). Notably, multiple genes in the wingless/Wnt (*tum, fz3, wls, Axn*) and Notch (*insv, Dl, krz*) signaling pathways were transcriptionally downregulated after puncture and trypsin puncture wounding.

### Identification of 8 Additional Localized Epidermal Wound Response Genes

In an effort to expand the small list of previously identified localized epidermal wound response genes, we selected 27 candidate genes that were significantly upregulated after trypsin puncture wounding. We chose these genes because they encode functions (mostly cell-cell signaling and transcription factors) that are known to be, or potentially involved in, regulatory pathways that control re-epithelialization, innate immunity, cell shape, and cell stress responses. Using *in situ* hybridization, these candidate genes were tested for wound-dependent epidermal transcriptional activation at one hour after puncture wounding of stage 15–17 wild-type embryos.

**Figure 9:**
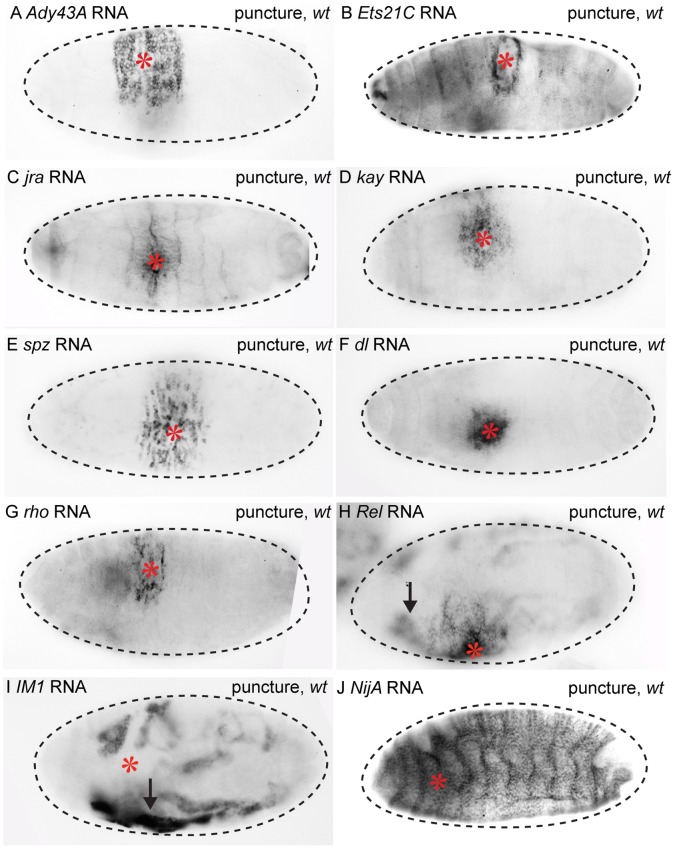
Novel localized epidermal, global epidermal, and fat body wound response genes in late stage *Drosophila* embryos. Alkaline phosphatase *in situ* hybridization with probes targeting RNA of candidate wound response genes. Puncture wounded and wild-type stage 15–17 embryos were compared for tissue-specific transcript induction one hour after wounding. (A) *Ady43A* transcripts accumulate in broad zone in the epidermis around puncture wound sites. (B) *Ets21C* transcripts are observably upregulated in the narrow zone wound site, and are already present in unwounded embryos at an easily detectable level throughout the entire epidermis. (C) An increase in *jra/jun* transcripts is observed in a narrow zone around the epidermal wound site. (D) An increase in *kay*/*fos* transcripts are also detected in a moderately broad zone around the epidermal wound site. (E) *Spz* transcripts are detected in a broad zone around the epidermal wound site. (F) After puncture wounding, *dorsal* transcripts are detected in a moderately broad zone around the epidermal wound site. (G) After puncture wounding, *rhomboid* transcripts are detected around the epidermal wound site. (H) After puncture wounding *Rel* RNA is upregulated around the epidermal wound site and in the fat body. (I) *IM1* RNA is upregulated throughout the fat body after puncture wounding, but not in the epidermis. (J) *NijA* RNA is upregulated throughout the entire epidermis after puncture wounding. Embryo bodies are outlined with dashed lines. The puncture wound site is indicated with a red asterisk. Black arrows point to fat body expression.

Eight of these 27 candidate genes were transcriptionally upregulated in epidermal cells surrounding the wound site. Thus, trypsin-mediated wounding allowed us to identify the following 8 additional localized epidermal wound response genes: *Ady43A (Ady43A), Ets at 21C (Ets21C), jun-related antigen (jra/jun), kayak (kay/fos), Relish (Rel), rhomboid (rho), spatzle (spz), and dorsal (dl)* (Figure 9). Many of these genes would not have been selected as candidates without the trypsin amplification results, as puncture-only microarray expression levels were not statistically significant at most time points for five of the eight genes tested (*jra/jun, kay/fos, spz, dl, rho*).

*Ady43A* transcripts are undetectable above background in unwounded embryos, but after puncture wounding transcripts accumulate in a broad zone of epidermal cells around wound sites (Figure 9A, Figure S7A). *Ets21C* transcripts are observed ubiquitously at low levels in the epidermis and in the ventral nerve cord in wild-type embryos; after puncture wounding *Ets21C* transcripts are detected at higher levels in a narrow zone of epidermal cells around wound sites (Figure 9B, Figure S7B). *Jra/jun* transcripts are detected at low levels in all cells of unwounded embryos, but after puncture wounding *jra/jun* transcripts are detected in a narrow zone of epidermal cells around wound sites (Figure 9C, Figure S7C). In unwounded embryos, *kay/fos* transcripts are detected at high levels in the late embryonic midgut & hindgut, and at low levels throughout the epidermis; after puncture wounding *kay/fos* transcripts are activated in a moderately broad zone of epidermal cells around wound sites (Figure 9D, Figure S7D). *Spz* RNA is expressed in the developing rectum of unwounded embryos; after puncture wounding, *spz* transcripts are also detected in a broad zone of epidermal cells around wound sites (Figure 9E, Figure S7E). Transcripts from *dl* are expressed weakly throughout the head and thoracic epidermis and other anterior tissues of unwounded embryos; after puncture wounding, *dl* transcripts are dramatically upregulated in a moderately broad zone of epidermal cells around wound sites (Figure 9F, Figure S7F). *Rho* RNA is detected in the peripheral nervous system of late stage unwounded embryos; after puncture wounding, *rho* transcripts are upregulated in a narrow zone of epidermal cells around wound sites (Figure 9G, Figure S7G). *Rel* RNA is expressed at low levels in the midgut and fat body of unwounded embryos; after puncture wounding, *Rel* transcripts are upregulated in a broad zone of epidermal cells around wound sites, as well as at higher levels in the fat body (Figure 9H, Figure S7H).

The remaining candidate genes we tested by *in situ* hybridization were not detectably upregulated in epidermal cells around wound sites. These were: *Ninjurin A, Diptericin, Attacin C, Dorsal-related immunity factor, Immune induced molecule 1, Immune induced molecule 2, Drosomycin, Tak1-like 1, slowpoke binding protein, p38c, Attacin A, locomotion defects, Hormone receptor-like in 38, cyclic-AMP response element binding protein A, faint sausage, Imaginal disc growth factor 1, Inositol 1,4,5-triphosphate kinase 1, Cecropin A1*, and *immune deficiency* (Figure S7K, L, unpublished data). However, several of these genes did show wound-dependent transcriptional activation in other tissues. For example, *Immune induced molecule 1 (IM1), Immune induced molecule 2, Dorsal-related immunity factor*, and *Rel* transcripts were upregulated above normal developmental expression levels in the fat body in response to clean puncture wounding (unpublished data, Figure 9I, H). In addition, *Ninjurin A (NijA)*, *Dorsal-related immunity factor, Diptericin,* and *Attacin C* were transcriptionally activated globally throughout the entire epidermis after clean puncture wounding (unpublished data, Figure 9J). These data suggest that clean puncture wounds produce systemic signals that can result in transcriptional activation for some genes in all cells of specific tissues; puncture wounds also produce signals that activate “epidermal wound gene” transcription only at short range; finally some genes like *Rel*, can transcriptionally respond to both short range “epidermal” signals, as well as systemic signals that impinge the fat body.

## Discussion

Our results indicate that a protease function is activated around embryonic puncture wound sites, and that serine protease activity is required to activate wound-induced transcription around wound sites. The injection of trypsin, at concentrations that do not detectably breach the epidermal paracellular barrier of *Drosophila* embryos, successfully mimics serine protease-dependent wound gene activation. By using trypsin to increasingly amplify the upregulation of genes that are normally activated after puncture wounding, we have obtained a deeper and richer view of the transcriptome regulated by epidermal wounding, adding considerably to the previous knowledge obtained by studies on the transcriptional response to localized epidermal wounds using needles or lasers in *Drosophila* embryos [6], [7], [14]–[16]. Eight of our newly defined wound-induced genes are transcriptionally activated in zones of epidermal cells around embryonic puncture wounds, and importantly, most of these localized upregulated genes include functions regulating either local epidermal innate immune signaling, re-epithelialization, EGF receptor signaling, or ETS-regulated transcriptional circuitry. In addition, clean puncture wounds also activate transcriptional responses remotely in the fat body, and other tissues, in a manner that suggests that tissue-specific cis-regulatory elements at different wound genes sense short and/or long range wound signals.

As previously shown with laser wounding of *Drosophila* embryos [15], we report that clean puncture wounding potently activates nearly the entire range of antimicrobial innate immune response genes that are mobilized to fight bacterial and fungal infection. In our wound protocol, puncturing very late-stage embryos is always prefaced by removal of the eggshell with bleach and repeated washes, which we believe results in a removal of almost all microbes prior to wounding, save those that are potentially incorporated between the vitelline membrane and the embryonic epidermis/developing cuticle. In the wild, puncture wounds are always associated with the entry of microbes. As suggested previously, the induction of a variety of innate immune genes after breaching epidermal barriers, even in the absence of microbes, would be evolutionarily selected to prime animals, whether vertebrate or invertebrate, to fight the inevitable entry of microbes via damaged barrier epithelia [15], [54]–[58]. The innate immune pathways activated specifically by clean wounds in the embryonic epidermis include genes from the Toll, Imd, and JNK pathways.

### Evolutionary Conservation of the Transcriptional Response to Epidermal Wounding

To gain a deeper understanding of the conservation of genes involved in the epidermal wound healing process, we compared the genes significantly upregulated on our *Drosophila* microarrays to previously published mammalian wound microarray profiles [59]–[62]. Twenty-seven *Drosophila* and mammalian genes are listed in Table 4 that are significantly upregulated after epidermal or general skin wounding treatments, and that are either orthologs or close structural relatives in the same gene family.

**Table 4:** Conservation of genes upregulated in response to clean epidermal wounding.

Fly gene symbol	Trypsin puncture up	Puncture up	Mammalian gene symbol	Reference	Notes
kay	Y	Y	FOS	Fitsialos et al. 2007; Cole et al. 2001; Cooper et al. 2004	
jra	Y	Y	JUN	Fitsialos et al. 2007; Cooper et al. 2004	
puc	Y	N	MKP-1	Cooper et al. 2004	
ple	Y	Y	TH	Cole et al. 2001	
Pvf2*	Y	Y	PDGF/VEGF	Fitsialos et al. 2007	*pvr not upregulated but ligand was upregulated
lox	N	Y	LOX	Colwell et al. 2008	
Rel	Y	Y	NFkB	Fitsialos et al. 2007	
cact	Y	Y	IkBalpha	Fitsialos et al. 2007: Cooper et al. 2004	
msn*	N	N	MAP4K4	Fitsialos et al. 2007; Pearson et al. 2009*	*msn upregulated at epidermal wound site
Rgk1	Y	Y	RRAD	Fitsialos et al. 2007	
nec	Y	Y	SERPINE1	Fitsialos et al. 2007	
MMP1	Y	N	MMP9	Fitsialos et al. 2007; Colwell et al. 2008; Cooper et al. 2004	closely related, not orthologs
Hsc70-1	Y	Y	CRYAB	Fitsialos et al. 2007	closely related, not orthologs
Cks85A	N	Y	CDK5R1	Fitsialos et al. 2007	closely related, not orthologs
PGRP-LF	Y	Y	LBP	Fitsialos et al. 2007	closely related, not orthologs
fs(1)N	Y	N	WDR33	Fitsialos et al. 2007	closely related, not orthologs
Socs44A	Y	N	SOCS1	Cole et al. 2001	
Ubi-p5E	N	Y	SIAHBP1	Cole et al. 2001	closely related, not orthologs
Rab6	Y	N	YPT3	Cole et al. 2001	closely related, not orthologs
Act42A	Y	N	ACTB	Cole et al. 2001	
crebA	Y	Y	CREBBP	Cole et al. 2001	closely related, not orthologs
loco	Y	Y	RGS12	Cole et al. 2001	
Ets21C	Y	Y	ETS1	Fitsialos et al. 2007	closely related, not orthologs
CalpA	Y	N	CAPN1	Cole et al. 2001	closely related, not orthologs
AnnIX	Y	Y	ANXA8	Cooper et al. 2004	closely related, not orthologs
Hr38	Y	Y	NR4A1	Cooper et al. 2004	
GstD1	Y	Y	GSTO1	Cooper et al. 2004	closely related, not orthologs

Twenty-seven genes that are transcriptionally upregulated after epidermal wounding in *Drosophila* and mammals. “Fly gene symbol” corresponds to Flybase gene symbol notation. Fly gene symbols listed are statistically significant (FDR<0.01) after puncture or trypsin puncture wounding for at least one timepoint (30, 60, or 120 minutes). The mammalian ortholog or the closest related mammalian protein to the listed fly gene is listed in the “Mammalian gene symbol” column. The listed mammalian genes were determined to be significantly upregulated after various wounding treatments. The “References” column lists the publications that identified the respective mammalian gene wound upregulation. “Trypsin puncture up” or “Puncture up” columns indicate if the corresponding fly gene was significantly upregulated (Y) or was not significantly upregulated (N) for at least one timepoint in the *Drosophila* microarrays.

Many mammalian wound microarray profiles have detected significant upregulation of FOS and JUN family genes, whose *Drosophila* orthologs were also significantly upregulated after embryonic puncture wounding [59]–[61] (Table 4). *Puckered*, a phosphatase that negatively regulates JNK signaling in *Drosophila* was significantly upregulated after embryonic trypsin puncture wounding (Table 4), as well as after larval and adult epidermal wounding [10], [11]. The mammalian ortholog of *puckered*, MKP-1 is also induced after incisional wounding of neonatal mouse epidermis [59]. Although *misshapen* (a *Drosophila* JNK kinase kinase kinase homolog) was not significantly upregulated in our *Drosophila* microarray experiments, *misshapen* transcription is locally upregulated in the epidermis surrounding puncture wound sites in *Drosophila* [6], [10], [11], and the mammalian *misshapen* ortholog, MAP4K4, is also upregulated after scratch wounding of keratinocytes [61].

The similarity of pathogen recognition, signaling pathways, and effector mechanisms of innate immunity in *Drosophila* and mammals indicates a common ancestry of some regulators and effectors of this defense system [63]. We found several genes associated with innate immune functions whose homologs were upregulated in both *Drosophila* and mammals after clean wounding. For instance, the *Drosophila* serpin *necrotic*, which negatively regulates the Toll innate immunity signaling pathway, and its mammalian ortholog SERPINE1, are significantly upregulated after *Drosophila* puncture wounding and keratinocyte scratch wounding assays, respectively [60], [61] (Table 4). Another gene that negatively regulates the Toll/Imd-mediated innate immune response, *cactus*, and its mammalian ortholog IκBα were significantly upregulated following *Drosophila* puncture wounding and mammalian wounding assays [59], [61] (Table 4). Additionally, both *Drosophila Relish* and its mammalian homolog NFκB, a conserved innate immunity transcription factor, were significantly upregulated following puncture and trypsin puncture wounding in *Drosophila* embryos, and scratch wounding of keratinocytes [61] (Table 4). Taken together, this is strong evidence that both the *Drosophila* and mammalian epidermis can mount an innate immune response after wounding, even in the absence of microbes.

One of the differences between the mammalian and *Drosophila* embryonic epidermal wound microarray profiles involved the expression of genes that regulate the cell cycle. Five cyclin genes were significantly downregulated after puncture and puncture plus trypsin wounding of *Drosophila* embryos (Table 3). However, keratinocyte scratch wounding heatmaps indicate that several cyclins (Cyclins E, F, G2) are significantly upregulated after wounding [61]. These results are consistent with previously published reports that *Drosophila* embryonic and larval wound healing events do not involve epidermal proliferation to close the wound gap, while mammalian keratinocytes at the wound margin actively proliferate behind the migrating epithelial wound-edge cells to re-epithelialize the barrier [35], [64]. Further support for the differences in cell proliferation induction levels is seen in the expression of *GADD45*. After puncture and trypsin puncture wounding, *Drosophila Gadd45* is upregulated in embryos, however human GADD45B is downregulated after keratinocyte scratch wounding [61] (Table 2). It has been reported that *GADD45*-induced G_2_-M arrest was associated with suppression of *GADD45*-mediated cell growth [65]. Collectively, this data suggests that puncture wounds are providing signals that instruct cells in the wounded *Drosophila* embryo not to divide, and to delay embryonic development until the wound is repaired. Additional evidence, from *in situ* hybridizations, for this idea is seen in the dramatic repression of transcript abundance for the *Drosophila* genes *Cyclin E* and *deoxyribonucleoside kinase* after clean puncture wounding of embryos (Figure S6). The differences in cell cycle gene expression levels seen on microarrays between mammals and *Drosophila* after wounding may simply reflect different sizes of the wounds, with typical mammalian wounds obliterating hundreds or thousands of cells, requiring cell division to replace the large number of missing cells. By contrast, puncture or laser wounding of *Drosophila* embryos or larvae involves the obliteration of only a few cells, which can be stitched together without proliferation [35]. The idea that different wound sizes can result in distinct gene expression responses is supported by the fact that large razor-inflicted wounds in the *Drosophila* adult epidermis do result in epidermal proliferation at a few cell diameters from the wound edge (Myungjin Kim and WM, unpublished data). It would be interesting to see if tiny mammalian skin wounds were repaired without proliferation, as they are in *Drosophila* embryos and larvae.

### Proposed Barrier Repair Roles for the Novel Localized Epidermal Wound Response Genes

The JNK signaling pathway is required for efficient wound healing in *Drosophila* adults [11]. *Puckered (puc*), a target of the JNK signaling pathway is induced at the epidermal wound edge and Jun Kinase is phosphorylated in wounded epidermal tissues [11]. In *kay/fos* mutant adults, *puc* reporter expression is no longer induced and in *kay/fos* and *jra/jun* mutant larvae there is a failure of the epidermal leading edge cells and more distal epidermal cells to elongate towards the wound edge, resulting in open wounds even 24 hours post-wounding [11], [13]. Considering all this, the *jra/jun* and *kay/fos* genes are presumably transcriptionally upregulated around embryonic epidermal wound sites to amplify JNK signaling events, which are required for re-epithelialization.

*Ets21C* has the potential of regulating the wound-dependent expression of other localized epidermal wound response genes given its function as a transcription factor. Previous studies have demonstrated that *Ets21C* is an immune regulated gene, although it was not known whether its activation was local or systemic in animals. Its expression can be induced in *Drosophila* S2 cells in response to an LPS challenge and this activation is dependent on activation of JNK signaling via the Imd pathway [66], [67]. A *Drosophila* antioxidant, *peroxiredoxin 5*, is involved in negative control of the immune response; *peroxiredoxin 5* regulates the dTak1-JNK arm of immune signaling and the downstream target gene *Ets21C* via its peroxidase activity [68]. Since JNK signaling is required for proper wound healing, *Ets21C* might also play a role in facilitating barrier repair and/or innate immunity after wounding since *Ets21C* can be regulated by JNK signaling.

Another signaling pathway that has been shown to regulate wound healing in *Drosophila* embryos is Epidermal Growth Factor Receptor (EGFR) signaling [21]. *EGFR* hypomorphic loss of function mutant embryos display wound closure defects 16 hours post-wounding [21]. *Rhomboid* is a transmembrane serine protease that promotes the intramembrane cleavage of Spitz, a *Drosophila* EGFR ligand [48]. It is reasonable to propose that *rhomboid* is transcriptionally upregulated at the localized epidermal wound site to amplify EGFR signaling via its serine protease function, assisting in the re-epithelialization of the wounded epidermis.

A *CecA1* (antimicrobial peptide gene) reporter can be globally activated in the embryonic fly epidermis after both clean and septic wounding and this activation requires the *imd* gene, an upstream regulator of *Relish* function [55], [69], [70]. The global epidermal expression of the *CecA1* reporter in the presence of microbial components required *Relish* function, adding evidence to the claim that the Imd innate immune signaling pathway plays a role in the epidermal expression of various antimicrobial peptides after septic injury. The localized upregulation of *Relish* gene expression that we observe around wound sites suggests that *Relish* is also in some manner contributing to barrier regeneration, although the targets it would regulate to achieve this function are currently unknown.

### Serine Protease Epidermal Wound Signal Activation

Perivitelline injection of trypsin is sufficient to induce a striking global epidermal wound response (Figure 3). From this, we propose that trypsin is capable of processing and activating an epidermal wound response ligand that is either attached extracellularly to epidermal cells or is present within the perivitelline space, ultimately initiating an epidermal wound response pathway. This is analogous to the way a serine protease cascade activates Toll receptor on the ventral side of very early *Drosophila* embryos after fertilization [26].

Previous work has shown that trypsin can process the pro-form of Spatzle in vitro into the active form of Spatzle that binds to the Toll receptor, as injection of trypsin-processed Spatzle into flies led to a strong induction of *Drosomycin*, a well-established target gene of the Toll pathway [71]. Thus, trypsin puncture wounding, as well as perhaps puncture-only wounding, might induce the Toll signaling pathway by activating *spatzle*, and explain why we observe such high induction levels of numerous innate immunity genes in the microarray profiles.

In horseshoe crab hemolymph, the Coagulogen protein gets converted into the clottable protein Coagulin via a serine protease cascade, followed by Coagulin homopolymerization and clot formation [72]. Interestingly, horseshoe crab Coagulogen has a three dimensional structure that is closely related to *Drosophila* Spatzle, suggesting that arthropod clot production and Toll-mediated innate immune signaling evolved from a common serine protease signaling system that activated signals for activating a transcriptional program to attack microbial infection, as well as for clotting [72]–[76]. Our results in *Drosophila* are consistent with the idea that epidermal wounds also trigger a similar serine protease cascade that activates epidermal repair transcriptional programs around wound sites.

## Materials and Methods

### *Drosophila* Stocks

The wild type strain used was *w^1118^. Grh^IM^*, *Ddc*.47 and *ple*-WE1 were previously described in [6], [7] and we refer to *Ddc*.47 and *ple*-WE1 as the *Ddc* and *ple* wound reporter lines in this paper.

### Puncture Wounding Treatment

Embryos were collected on apple juice agar plates and aged to 15–17 h at 25°C. Embryos were washed into mesh baskets, dechorionated in bleach for 1 min, then washed copiously with water. Embryos were then transferred to a clean slab of apple juice agar and aligned for 30–60 min at 18°C, transferred to slides with double-sided tape, then covered in a 1∶1 ratio of 700∶27 weight halocarbon oil. Embryos were then wounded bilaterally with fresh microinjection needles made from an automated puller mounted on a micromanipulator, allowed to recover for 5–6 h at room temperature or overnight at 4°C, and then visualized under fluorescent light in a compound microscope to determine wound reporter activity. At least 3 independent experiments with at least 30 successfully wounded embryos were performed. Assays involving homozygous deletion or mutant embryos were performed in parallel to heterozygous-balancer embryos. A Kr-GFP fluorescent marker on the balancer chromosome, was used to determine the genotype of the embryos [77]. All embryos were impaled using a micromanipulator so that the needle protruded 1 embryo-width from the exit wound. Wound reporter responses were rated on a scale of “no activity, localized activity, or global activity.” Images were obtained by wounding embryos with microinjection needles and imaged on a Leica SP2 confocal microscope, selecting representative embryos to image. Images were resized while constraining proportions to maintain resolution. Adobe Photoshop adjustment functions were used equally on images to enhance clarity, but not to obscure, eliminate, or misrepresent any information. Original images are available on request.

### Puncture Wounding Injections

Individual embryos were simultaneously wounded and injected by using a syringe to expel the various solutions into the body cavity of the embryo. A Pipetman was used to load the solutions to be injected into the pulled capillary microinjection needles. Needles were broken on the side of a glass cover slip on a glass slide. Serine Protease-Trypsin from bovine pancreas was solubilized in 1 mM HCl pH 3.0 to 2 mg/mL or 83 µM (Sigma). Hydrogen Peroxide was diluted in water to 0.6 M (Fisher). Pefabloc SC was diluted in water to 91 mM (Roche). Marimastat (Tocris) was diluted in DMSO to 100 mM. Papain was diluted in water to 10 mg/mL (Sigma). Proteinase K was diluted in water to 1 mg/mL (Sigma). Chemical-wounded embryos were simultaneously wounded and injected with a 1∶4 ratio of 1% toluidine blue dye and solubilized compounds. Toluidine blue dye allowed for visual confirmation of solubilized compounds being injected into the body cavity. Control embryos were wounded with a broken needle containing 1∶4 ratios of 1% toluidine blue dye and solute without chemical. A wide range of chemical concentrations was tested to obtain optimal activation of the epidermal wound reporter and maintain high levels of embryo survival after body cavity injection.

### Perivitelline Injections

Embryos were dechorionated, aligned on a slide and dehydrated using desiccant for 30–45 minutes, then a 1∶4 ratio of 1% toluidine blue dye and 10 mg/mL of 70,000 MW Rhodamine B isothiocyanate-Dextran (Sigma, St. Louis, MO, R9379) was injected into the perivitelline space either with water or 1 mM HCl carrier solutions or with chemical. Embryos were allowed to recover for 5–6 h at room temperature or overnight at 4°C, and then visualized with a confocal microscope to determine wound reporter activity.

### Survivability Assay

After wounding, embryos were put in a humidity chamber overnight at 18°C. The next morning living, hatched larvae were transferred to a food vial containing yeast and allowed to progress through development to adulthood at 18°C for scoring.

### *Drosophila* Microarray Sample Collection

The following *Drosophila* embryo collection procedures were carried out in duplicate. Wild-type embryos were crushed in Trizol and stored at −80°C until multiple samples could be pooled for each treatment (unwounded, puncture wounded or trypsin puncture wounded) and time point (30, 60 or 120 minutes post-wounding). Approximately 500 wild-type embryos were collected for each treatment and time point and stored frozen in Trizol. Embryos were ground in Trizol using a pestle, RNA was purified using standard Trizol procedures. Total RNA was further purified with Qiagen’s RNeasy Mini Kit. RNA integrity was assessed with the Agilent Bioanalyzer.

### Microarray Design and Analysis

Predesigned *Drosophila melanogaster* arrays were ordered from Agilent (Design ID # 18972). A total of 43,603 features were printed on each chip, which mapped to ∼13,000 unique FlyBase genes. Intensity values from redundant probes (or unique probes targeting the same gene) were grouped, and only the highest fold-change values were used in these analyses. RNA labeling, hybridizations, intensity quantification, data normalization, FDR calculations, and GO annotations were carried out by the Biogem Core facility (UC San Diego); see Text S1 for an in-depth description of the microarray analyses. Manual *Drosophila* gene classifications (Tables 2 and 3) were carried out by consulting Flybase (www.flybase.org) [78] and The Gene Ontology (www.geneontology.org), as well as with literature searches. The normalized microarray results have been deposited in the European Molecular Biology Laboratory European Bioinformatics Institute in the ArrayExpress database (www.ebi.ac.uk/arrayexpress/), and the accession number for the *Drosophila* datasets is E-MEXP-3755.

### Alkaline Phosphatase *in situ* Hybridization

Wild-type stage 15–17 embryos were puncture wounded and then allowed to recover for one hour before fixation. Unwounded wild-type stage 15–17 embryos were used as a control for developmental expression of each candidate probe (unpublished data). The enzymatic developing reaction times were identical for unwounded and puncture wounded embryos with respect to the given probe. Full-length cDNA clones for candidate gene RNA probe synthesis were obtained from the Drosophila Genomics Resource Center supported by NIH grant OD010949-10. Each probe incorporated digoxygenin (DIG) labeled nucleotides conjugated to alkaline phosphatase. NBT/BCIP substrate was used to detect tissue-specific gene expression of each probe. Enzymatic reaction times ranged from 15 minutes to one hour, depending on the probe. *Ddc*-DIG probes were used as a positive control for localized staining around the puncture wound sites in the epidermis. To stop the enzymatic developing reaction, embryos were washed 3 times in 1X PBT and mounted in DTG before they were imaged using a Leica light microscope.

### Immunostaining

Fixed wild-type embryos were washed in phosphate buffered saline with Tween (PBTwx), then incubated in a blocking solution of PBTwx with Western blocking reagent (WBR; Roche) for 30 minutes at room temperature. Incubations with primary antibodies were performed in PBTwx+WBR at 4°C overnight, and incubations with fluorescently labeled secondary antibodies were performed in PBTwx+WBR at room temperature for 2 hours. Primary antibody mouse anti-Fasciclin 3 (7G10 concentrate, from the Developmental Studies Hybridoma Bank) was used at a 1∶200 dilution. The fluorescently labeled secondary antibody from Invitrogen (Alexa Fluor 488 donkey anti-mouse IgG) was used at 1∶400 dilution. Embryos were mounted in DTG. All fluorescent images were collected using a Leica SP2 laser-scanning confocal microscope, with identical instrument settings (at non-saturated gain levels) for both experimental and control samples. Optical sections were scanned at 1 µm thicknesses, and maximum-projection images are shown.

### Fluorescent *in situ* Hybridization

*Ddc* and *ple* probes were generated from partial or full cDNA clones from the *Drosophila* Gene Collection. Probe labeling and hybridization protocol was as described in Kosman et al. [79].

### Necrosis Staining

Wild-type or *Ddc*.47 stage 15–17 embryos were wounded and then allowed to recover for 2 hours at room temperature. Embryos were rinsed off slides with heptane and then put into a scintillation vial with 1∶1 heptane: 1X PBS. Embryos were shaken at 250 RPM for 5 minutes on a gyrotory shaker. Embryos at interface were removed and washed with 1X PBS. The Apoptotic and Necrotic and Healthy Cells Quantification Kit (Biotium, Inc., catalog # 30018) was used to visualize necrosis with Ethidium homodimer III. Stained embryos were placed on a slide with 700 Halocarbon oil and a coverslip was added before immediately imaging with a SP2 Leica confocal microscope.

### Apoptosis Staining

Wild-type stage 15–17 embryos were wounded and then allowed to recover for 2 hours at RT. Unwounded controls were present at RT for 2 hours, as well. Embryos were rinsed off slides with heptane and put in a scintillation vial with 1∶1 heptane: 1.6×10–6 M Acridine Orange in 1X PBS. Embryos were shaken at 250RPM for 5 minutes on a gyrotory shaker. Embryos were removed from the interface and rinsed 3 times in 1X PBS. Embryos were placed on a slide and mounted in 700 Halocarbon oil and a coverslip was added before being immediately imaged with a Leica SP2 confocal microscope.

### Quantitative RT-PCR

Eleven genes that exhibited variable levels of upregulation (low, medium, high) in response to puncture and trypsin wounding on the microarray platform, were validated with qRT-PCR testing. Primers for *IM1, IM2, AttB, CecA1, Drs, Mtk, DptB*, *PGRP-LB,* CG9733, *ple, Rel,* and *rpd49* were designed with the Roche Universal Probe Library. Primer testing generated standard curves for each set of primers, but only primers with an efficiency of 90% or greater and a single melting curve peak were used for relative quantitation runs. RNA from unwounded, puncture wounded, and trypsin wounded wild-type embryos was subjected to reverse transcription using Retroscript (Ambion) and the resulting cDNA was quantified by qRT-PCR with SYBR Green. Gene expression was normalized using *rpd49* as an endogenous control. Fold change values were generated by using unwounded levels as baseline expression.

### Bovine Serum Albumin-Green Wounding

Wild-type stage 15–17 embryos were puncture wounded with needles filled with DQ Green BSA (Molecular Probes) that was solubilized in 1X PBS. Embryos were either wounded with 1X PBS or 2 mg/mL DQ Green BSA in 1X PBS or puncture wounded without solution. Embryos were mounted in 700 Halocarbon oil, a cover slip was added, and imaged 30 minutes post-wounding to observe any signal emitted at 515 nm with a Leica SP2 confocal microscope.

## Supporting Information

Figure S1 ***Ddc*** **.47 global epidermal activation is largely serine protease-specific**. Confocal images of *Ddc*.47 embryos wounded with various chemical proteases or protease inhibitors. (A) Control water puncture wounded embryos only activate *Ddc*.47 localized to the wound site. (B) Proteinase K puncture wounded embryos activate the reporter globally throughout the epidermis**.** (C) Control water wounded embryos activate *Ddc*.47 locally around the wound site. (D) Papain puncture wounded embryos exhibit weak, patchy expanded epidermal reporter activation. (E) Control DMSO puncture-wounded embryos only activate the wound reporter at the wound site. (F) A similar level of localized *Ddc*.47 reporter activation is observed in Marimastat puncture wounded embryos. Arrows mark the wound site. Dashed lines in the data panels mark the outlines of embryos. *Ddc*.47 is a fluorescent reporter that includes a wound-induced DNA enhancer from the *Ddc* locus.
(TIF)

Figure S2 **Trypsin treatment does not disrupt Fasciclin III epidermal cell junction morphology**. Confocal images of late stage wild-type embryos stained with mouse anti-Fasciclin III (FasIII) protein. (A) Untreated wild-type embryos exhibit FasIII staining at epidermal cell membrane junctions. (B) Embryos puncture wounded with trypsin exhibit FasIII staining at epidermal cell membrane junctions. Arrow mark wound site. Dashed lines in the data panels mark the outline of embryos.
(TIF)

Figure S3 **Hydrogen peroxide is sufficient to induce widespread** ***Ddc*** **.47 activation in the absence of puncture wounding.** Confocal images of *Ddc*. 47 (green) embryos injected with fluorescent Rhodamine Dextran (red) to assess epidermal integrity and reporter activation after hydrogen peroxide perivitelline injection. (A, B) Perivitelline injection of hydrogen peroxide along with Rhodamine Dextran globally activates the *Ddc*.47 wound reporter without compromising the epidermal barrier since Rhodamine Dextran in contained within the perivitelline space. Arrows mark the wound site. Dashed lines in the data panels mark the outlines of embryos. *Ddc*.47 is a fluorescent reporter that includes a wound-induced DNA enhancer from the *Ddc* locus.
(TIF)

Figure S4 **Microarray scatterplots and rankings of puncture and trypsin puncture wounding treatments**. (A) A scatterplot of log2 (puncture/unwound) replicate 1 vs. log2 (puncture/unwound) replicate 2 at the 30 minute time point forms a largely diagonal line, indicating consistency between the biological replicate samples. (B) The same was done for both trypsin puncture wounded replicates (trypsin puncture/unwound) at the 30 min time points, resulting in a similar diagonal linear relationship, indicating a tight correlation of the two biological replicates. (A, B) Ranking of the genes in replicates 1 and 2 show a bunching of points near left lower end and right upper end, indicating that the genes preserve their ranking in the two replicates of both wounding treatments. The False Discovery Rate (FDR) was calculated from these ranks using F statistics assuming constant variance in log space.
(TIF)

Figure S5 **Quantitative RT-PCR validation of microarray upregulated fold change values.** Quantitative RT-PCR was carried out on a selection of 11 upregulated puncture and trypsin puncture genes (*IM2, IM1, AttB, CecA1, Drs, Mtk, DptB, PGRP-LB,* CG9733, *ple, Rel*) on the *Drosophila* microarray. Genes were chosen to span a wide range of fold changes. Error bars depict standard deviation between replicate treatments. (A) The qPCR results verify the directionality of the puncture wound fold changes (puncture/unwound) seen on the microarrays, as well as (in most cases) the approximate fold change values. (B) The qPCR results verify the directionality of the trypsin puncture wound fold changes (trypsin puncture/unwound) seen on the microarrays, as well as (in most cases) the approximate fold change values. Results were analyzed using the housekeeping gene *rp49* (CG7939) as a control. Primer sequences were as follows: *IM2* - tcgtcaccgtctttgtgttc and cagtccccgttgattaccac; *IM1* - gtttttgtgctcggtctgc and tgatcacatttcctggatcg; *AttB* - caaccataatgtggtaggtcagg and gtgtgtgttttggtcaaagagg; *CecA1* - gaagctgggtggctgaag and attgtggcatcccgagtg; *Drs* - ttcgctgtcctga and acagggacccttgtatcttcc; *Mtk* - tcttggagcgatttttctgg and tctgccagcactgatgtagc; *DptB* - ctgcagcctgaaccactg and cttgctttgggcttccac; *PGRP-LB* - tgatcggagattggagaacc and cccttgaaaacgccaaag; CG9733 - gaacgggaagtcggaacac and atctagcccaaac; *ple* - cgccatcaagaaatcctacag and ctcgaaacgggcatcatc; *Rel* - aatagagacacgctcctgcac and ggccagcttcagtttgtcc; *rp49* - tcggatcgatatgctaagctg and cgacgcactctgttgtcg.
(TIF)

Figure S6 ***In situ*** **hybridization validation of downregulated microarray gene expression.** Alkaline phosphatase *in situ* hybridization with probes targeting RNA of two downregulated genes from the microarray. Wild-type stage 15–17 embryos were examined for transcriptional expression of *CycE* and *dnk* during late embryogenesis. (A) Embryos express *CycE* transcripts throughout the ventral nerve cord and brain tissues during late-stage embryogenesis. (B) After puncture wounding, *CycE* transcripts are only faintly detected in the brain. (C) Embryos express *dnk* transcripts throughout the midgut, ventral nerve cord, brain, anal pads, and caecum during late-stage embryogenesis. (D) After puncture wounding, *dnk* transcripts are no longer detected in these tissues. Arrows denote the site of puncture wounds. Dashed lines outline the embryos.
(TIF)

Figure S7 **Developmental expression of novel epidermal wound response genes in late-stage** ***Drosophila*** **embryos.** Alkaline phosphatase *in situ* hybridization with probes targeting RNA of candidate wound response genes. Wild-type stage 15–17 embryos were examined for tissue-specific transcriptional expression during late embryogenesis. (A) *Ady43A* transcripts are undetected in unwounded embryos. (B) *Ets21C* transcripts are observably detectable at low levels throughout the entire epidermis and in the ventral nerve cord. (C) *Jra/jun* transcripts are expressed at low levels throughout the epidermis. (D) *Kay/fos* transcripts are detected in the midgut and hindgut and at low levels throughout the epidermis. (E) *Spz* transcripts are detected in the developing rectum (F) *Dorsal* transcripts are detected weakly throughout the head and thoracic epidermis and other anterior tissues. (G) *Rhomboid* transcripts are detected in the peripheral nervous system. (H) *Rel* RNA is expressed at low levels in the midgut and fat body. (I) *IM1* RNA expressed at very low levels throughout the epidermis. (J) *NijA* RNA is expressed at low levels throughout the epidermis. (K) *Takl1* RNA is expressed in the midgut and hindgut in unwounded embryos. No *takl1* transcripts are detected at the epidermal wound site. (L) *CrebA* transcripts are detected in the salivary glands. *CrebA* is not detected at the epidermal wound site in puncture wounded embryos. Embryo bodies are outlined with dashed lines. The puncture wound site is indicated with an arrow.
(TIF)

Table S1 **Trypsin concentration effects wound response activation levels and survival.** Increasing the concentration of trypsin increases the percentage of *Ddc*.47 embryos exhibiting global epidermal reporter activation and decreases the percentage of *Ddc*.47 embryos that hatch as larvae. The same number of embryos that hatched were able to survive to adulthood. On average, injection of the trypsin carrier solution resulted in no embryos activating global wound reporter activation (WRA) and 12% of the embryos did not hatch (N = 787). We believe the 12% non-hatching is largely attributed to non-fertilization, or developmental defects. Trypsin-induced death percentages were calculated by subtracting the percentage of trypsin wounded embryos that died from the percentage of trypsin carrier solution wounded embryos that appeared to be unfertilized. Number (#) of embryos was calculated by subtracting the percentage of embryos that appeared to be unfertilized from the total number of embryos wounded in each trypsin enzyme concentration treatment. *Ddc*.47 is a fluorescent reporter that includes a wound-induced DNA enhancer from the *Ddc* locus.
(PDF)

Table S2 **Trypsin-puncture wounding further increases the upregulation of puncture-only upregulated genes.** The fold changes of the 210 significantly upregulated genes after puncture wounding at the 120 minute timepoint were compared to their fold change after trypsin puncture wounding at 120 minutes. The 120 minute timepoint was used for comparison since this timepoint contained the highest amount of upregulated genes after either wounding treatment. “CG #” refers to the accession numbers from Flybase. “Gene symbol” refers to the gene symbol on Flybase. “Puncture fold change” refers to fold changes seen in expression values after puncture wounding relative to wild-type untreated values. “Trypsin fold change” refers to fold changes seen in gene expression values after trypsin puncture wounding relative to wild-type untreated values. “Highest fold change” refers to whether puncture or trypsin puncture wounding resulted in the highest fold change for the corresponding gene. #N/A indicates that the trypsin wounding treatment did not result in a significant fold change value (FDR>0.01).
(PDF)

Text S1 **Statistical and Bioinformatical Analyses of Microarray Data.** Below is a detailed explanation of how statistically significant genes from the *Drosophila* microarray sets were determined.
(DOC)
