# Supplementary figures and images for: Serine Proteolytic Pathway Activation Reveals an Expanded Ensemble of Wound Response Genes in Drosophila
Source: PLoS One. 2013 Apr 24;8(4):e61773. doi: 10.1371/journal.pone.0061773 (PMC3634835; doi:10.1371/journal.pone.0061773)

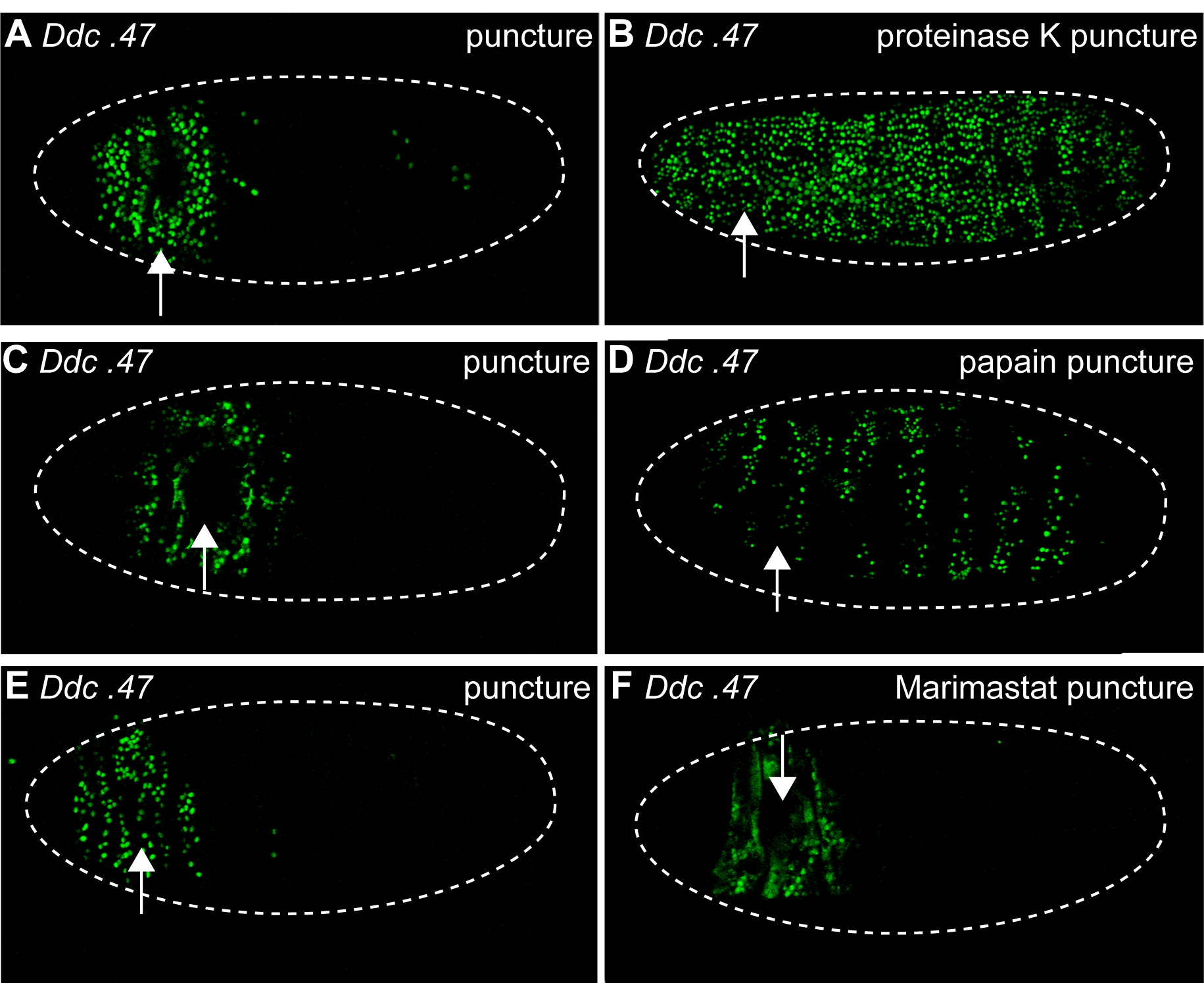

Supplement: Figure S1 — Ddc .47 global epidermal activation is largely serine protease-specific. Confocal images of Ddc.47 embryos wounded with various chemical proteases or protease inhibitors. (A) Control water puncture wounded embryos only activate Ddc.47 localized to the wound site. (B) Proteinase K puncture wounded embryos activate the reporter globally throughout the epidermis. (C) Control water wounded embryos activate Ddc.47 locally around the wound site. (D) Papain puncture wounded embryos exhibit weak, patchy expanded epidermal reporter activation. (E) Control DMSO puncture-wounded embryos only activate the wound reporter at the wound site. (F) A similar level of localized Ddc.47 reporter activation is observed in Marimastat puncture wounded embryos. Arrows mark the wound site. Dashed lines in the data panels mark the outlines of embryos. Ddc.47 is a fluorescent reporter that includes a wound-induced DNA enhancer from the Ddc locus. (TIF) [file pone.0061773.s001.tif]

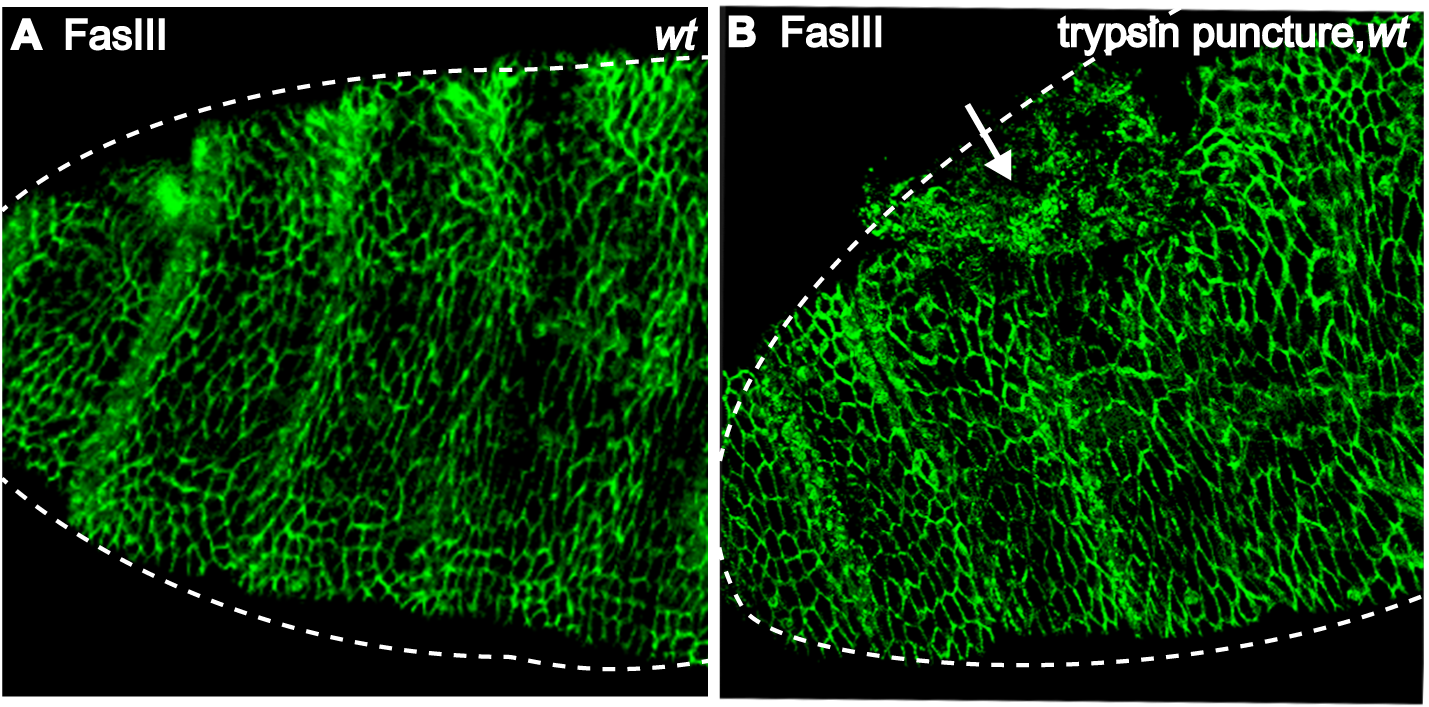

Supplement: Figure S2 — Trypsin treatment does not disrupt Fasciclin III epidermal cell junction morphology. Confocal images of late stage wild-type embryos stained with mouse anti-Fasciclin III (FasIII) protein. (A) Untreated wild-type embryos exhibit FasIII staining at epidermal cell membrane junctions. (B) Embryos puncture wounded with trypsin exhibit FasIII staining at epidermal cell membrane junctions. Arrow mark wound site. Dashed lines in the data panels mark the outline of embryos. (TIF) [file pone.0061773.s002.tif]

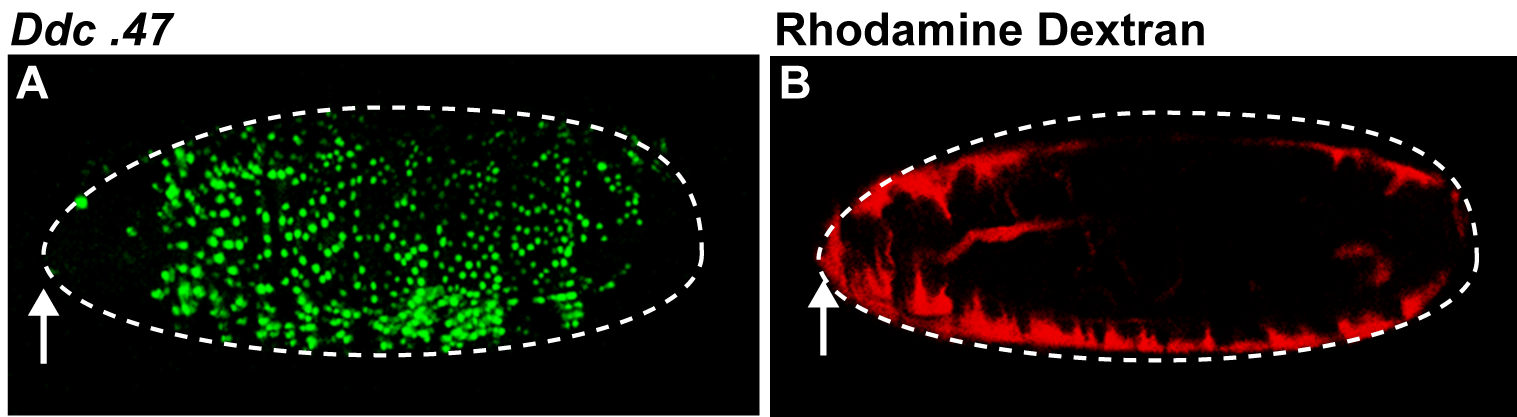

Supplement: Figure S3 — Hydrogen peroxide is sufficient to induce widespread Ddc .47 activation in the absence of puncture wounding. Confocal images of Ddc. 47 (green) embryos injected with fluorescent Rhodamine Dextran (red) to assess epidermal integrity and reporter activation after hydrogen peroxide perivitelline injection. (A, B) Perivitelline injection of hydrogen peroxide along with Rhodamine Dextran globally activates the Ddc.47 wound reporter without compromising the epidermal barrier since Rhodamine Dextran in contained within the perivitelline space. Arrows mark the wound site. Dashed lines in the data panels mark the outlines of embryos. Ddc.47 is a fluorescent reporter that includes a wound-induced DNA enhancer from the Ddc locus. (TIF) [file pone.0061773.s003.tif]

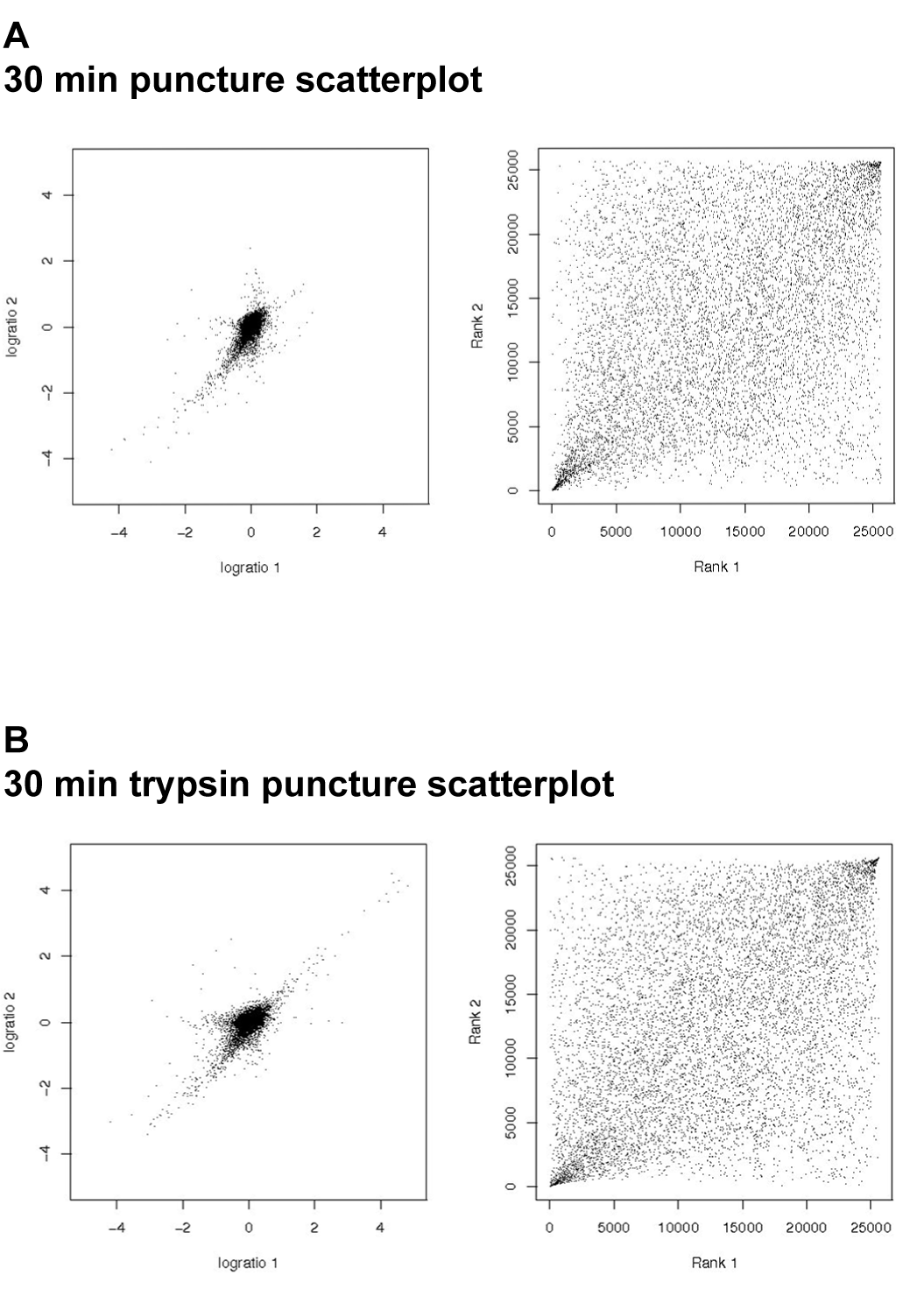

Supplement: Figure S4 — Microarray scatterplots and rankings of puncture and trypsin puncture wounding treatments. (A) A scatterplot of log2 (puncture/unwound) replicate 1 vs. log2 (puncture/unwound) replicate 2 at the 30 minute time point forms a largely diagonal line, indicating consistency between the biological replicate samples. (B) The same was done for both trypsin puncture wounded replicates (trypsin puncture/unwound) at the 30 min time points, resulting in a similar diagonal linear relationship, indicating a tight correlation of the two biological replicates. (A, B) Ranking of the genes in replicates 1 and 2 show a bunching of points near left lower end and right upper end, indicating that the genes preserve their ranking in the two replicates of both wounding treatments. The False Discovery Rate (FDR) was calculated from these ranks using F statistics assuming constant variance in log space. (TIF) [file pone.0061773.s004.tif]

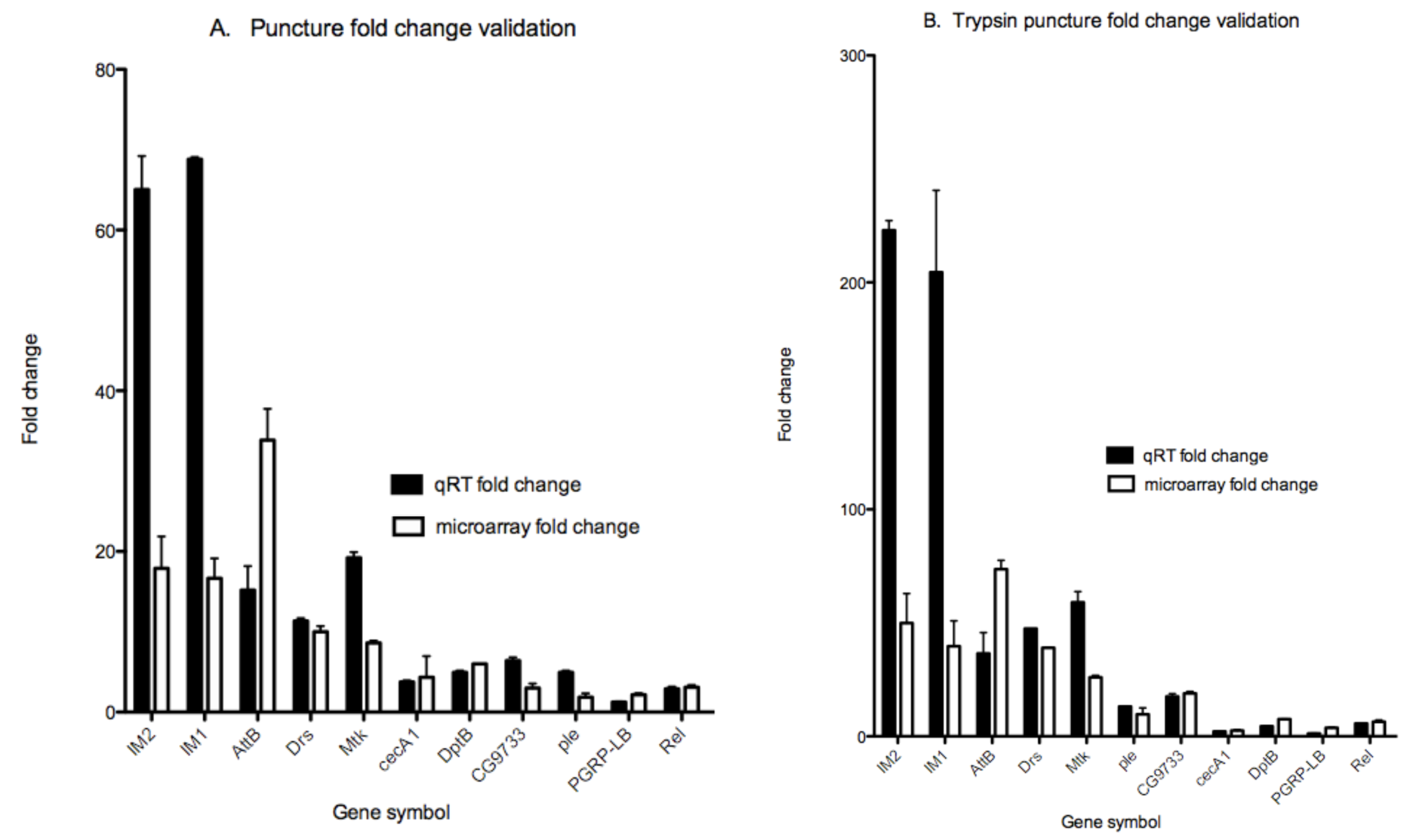

Supplement: Figure S5 — Quantitative RT-PCR validation of microarray upregulated fold change values. Quantitative RT-PCR was carried out on a selection of 11 upregulated puncture and trypsin puncture genes (IM2, IM1, AttB, CecA1, Drs, Mtk, DptB, PGRP-LB, CG9733, ple, Rel) on the Drosophila microarray. Genes were chosen to span a wide range of fold changes. Error bars depict standard deviation between replicate treatments. (A) The qPCR results verify the directionality of the puncture wound fold changes (puncture/unwound) seen on the microarrays, as well as (in most cases) the approximate fold change values. (B) The qPCR results verify the directionality of the trypsin puncture wound fold changes (trypsin puncture/unwound) seen on the microarrays, as well as (in most cases) the approximate fold change values. Results were analyzed using the housekeeping gene rp49 (CG7939) as a control. Primer sequences were as follows: IM2 - tcgtcaccgtctttgtgttc and cagtccccgttgattaccac; IM1 - gtttttgtgctcggtctgc and tgatcacatttcctggatcg; AttB - caaccataatgtggtaggtcagg and gtgtgtgttttggtcaaagagg; CecA1 - gaagctgggtggctgaag and attgtggcatcccgagtg; Drs - ttcgctgtcctga and acagggacccttgtatcttcc; Mtk - tcttggagcgatttttctgg and tctgccagcactgatgtagc; DptB - ctgcagcctgaaccactg and cttgctttgggcttccac; PGRP-LB - tgatcggagattggagaacc and cccttgaaaacgccaaag; CG9733 - gaacgggaagtcggaacac and atctagcccaaac; ple - cgccatcaagaaatcctacag and ctcgaaacgggcatcatc; Rel - aatagagacacgctcctgcac and ggccagcttcagtttgtcc; rp49 - tcggatcgatatgctaagctg and cgacgcactctgttgtcg. (TIF) [file pone.0061773.s005.tif]

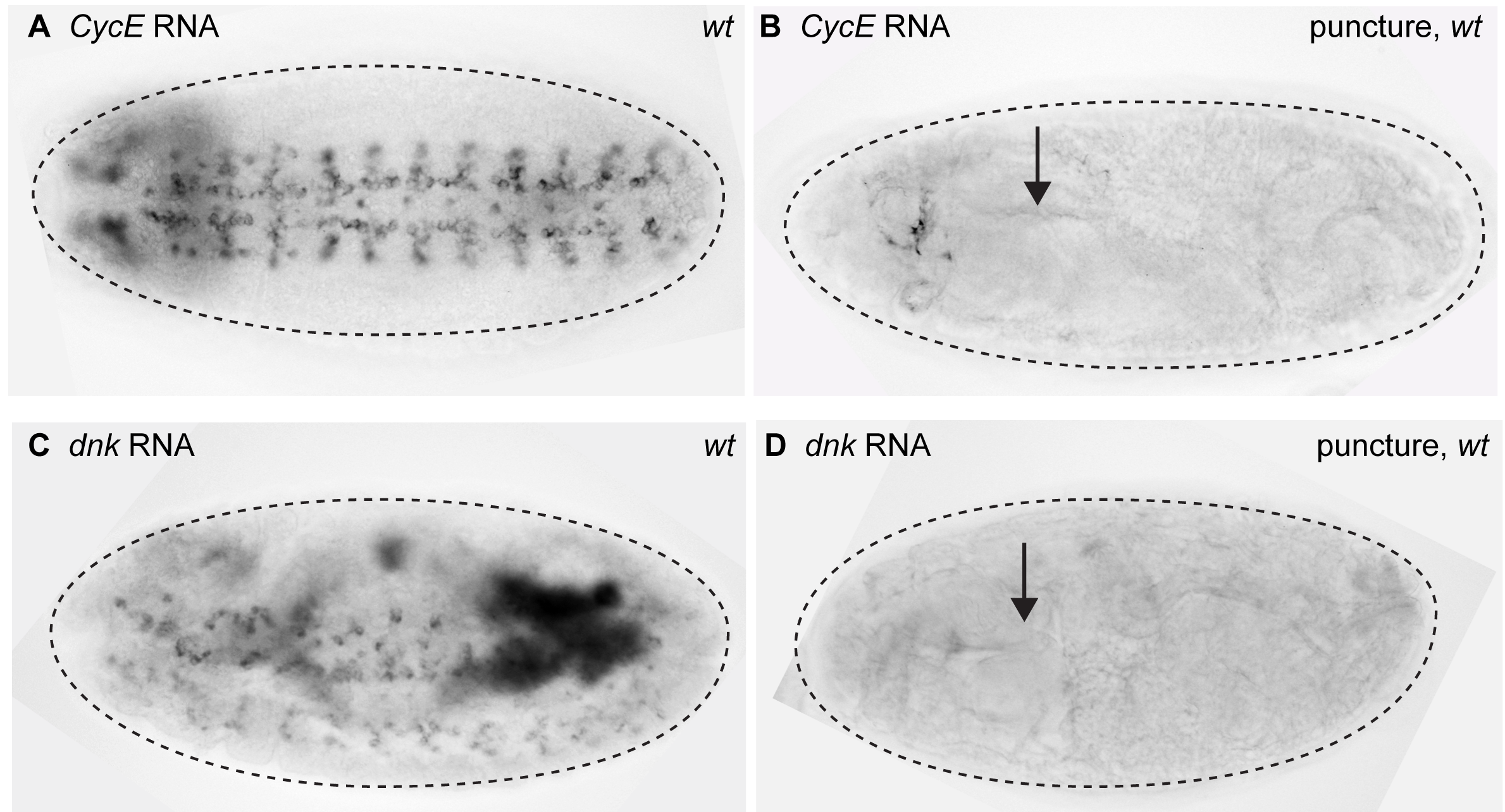

Supplement: Figure S6 — In situ hybridization validation of downregulated microarray gene expression. Alkaline phosphatase in situ hybridization with probes targeting RNA of two downregulated genes from the microarray. Wild-type stage 15–17 embryos were examined for transcriptional expression of CycE and dnk during late embryogenesis. (A) Embryos express CycE transcripts throughout the ventral nerve cord and brain tissues during late-stage embryogenesis. (B) After puncture wounding, CycE transcripts are only faintly detected in the brain. (C) Embryos express dnk transcripts throughout the midgut, ventral nerve cord, brain, anal pads, and caecum during late-stage embryogenesis. (D) After puncture wounding, dnk transcripts are no longer detected in these tissues. Arrows denote the site of puncture wounds. Dashed lines outline the embryos. (TIF) [file pone.0061773.s006.tif]

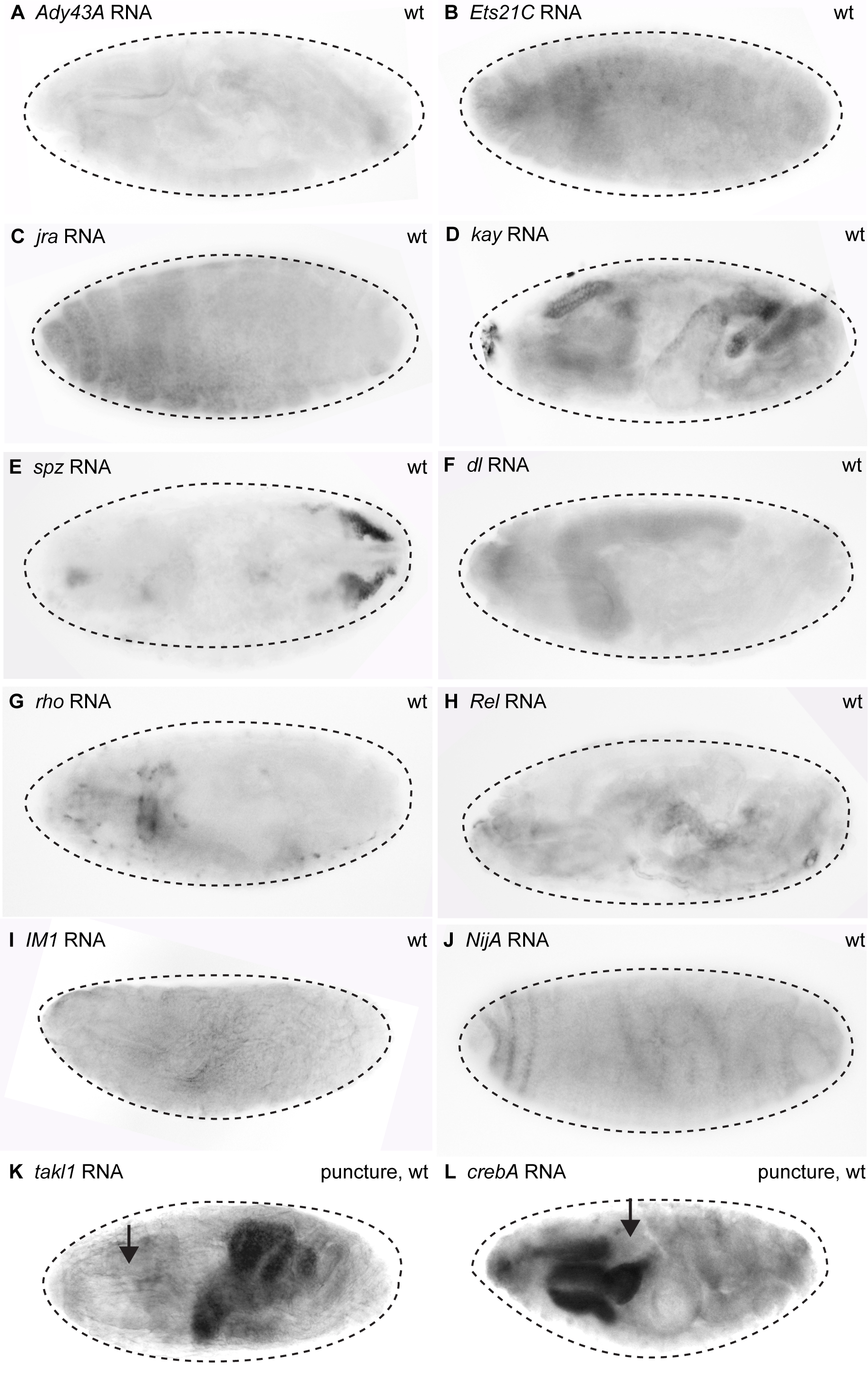

Supplement: Figure S7 — Developmental expression of novel epidermal wound response genes in late-stage Drosophila embryos. Alkaline phosphatase in situ hybridization with probes targeting RNA of candidate wound response genes. Wild-type stage 15–17 embryos were examined for tissue-specific transcriptional expression during late embryogenesis. (A) Ady43A transcripts are undetected in unwounded embryos. (B) Ets21C transcripts are observably detectable at low levels throughout the entire epidermis and in the ventral nerve cord. (C) Jra/jun transcripts are expressed at low levels throughout the epidermis. (D) Kay/fos transcripts are detected in the midgut and hindgut and at low levels throughout the epidermis. (E) Spz transcripts are detected in the developing rectum (F) Dorsal transcripts are detected weakly throughout the head and thoracic epidermis and other anterior tissues. (G) Rhomboid transcripts are detected in the peripheral nervous system. (H) Rel RNA is expressed at low levels in the midgut and fat body. (I) IM1 RNA expressed at very low levels throughout the epidermis. (J) NijA RNA is expressed at low levels throughout the epidermis. (K) Takl1 RNA is expressed in the midgut and hindgut in unwounded embryos. No takl1 transcripts are detected at the epidermal wound site. (L) CrebA transcripts are detected in the salivary glands. CrebA is not detected at the epidermal wound site in puncture wounded embryos. Embryo bodies are outlined with dashed lines. The puncture wound site is indicated with an arrow. (TIF) [file pone.0061773.s007.tif]
