# Supplementary material for: Serine Proteolytic Pathway Activation Reveals an Expanded Ensemble of Wound Response Genes in Drosophila
Source: PLoS One. 2013 Apr 24;8(4):e61773. doi: 10.1371/journal.pone.0061773 (PMC3634835; doi:10.1371/journal.pone.0061773)
Supplement: Table S1 — Trypsin concentration effects wound response activation levels and survival. Increasing the concentration of trypsin increases the percentage of Ddc.47 embryos exhibiting global epidermal reporter activation and decreases the percentage of Ddc.47 embryos that hatch as larvae. The same number of embryos that hatched were able to survive to adulthood. On average, injection of the trypsin carrier solution resulted in no embryos activating global wound reporter activation (WRA) and 12% of the embryos did not hatch (N = 787). We believe the 12% non-hatching is largely attributed to non-fertilization, or developmental defects. Trypsin-induced death percentages were calculated by subtracting the percentage of trypsin wounded embryos that died from the percentage of trypsin carrier solution wounded embryos that appeared to be unfertilized. Number (#) of embryos was calculated by subtracting the percentage of embryos that appeared to be unfertilized from the total number of embryos wounded in each trypsin enzyme concentration treatment. Ddc.47 is a fluorescent reporter that includes a wound-induced DNA enhancer from the Ddc locus. (PDF) [file pone.0061773.s008.pdf]

[Trypsin] uM	Global WRA	Trypsin-induced Death	# Embryos
31	13%	18%	97
40	56%	20%	142
52	77%	59%	149
83	78%	60%	174
233	85%	79%	153
