# Supplementary material for: Serine Proteolytic Pathway Activation Reveals an Expanded Ensemble of Wound Response Genes in Drosophila
Source: PLoS One. 2013 Apr 24;8(4):e61773. doi: 10.1371/journal.pone.0061773 (PMC3634835; doi:10.1371/journal.pone.0061773)
Supplement: Table S2 — Trypsin-puncture wounding further increases the upregulation of puncture-only upregulated genes. The fold changes of the 210 significantly upregulated genes after puncture wounding at the 120 minute timepoint were compared to their fold change after trypsin puncture wounding at 120 minutes. The 120 minute timepoint was used for comparison since this timepoint contained the highest amount of upregulated genes after either wounding treatment. “CG #” refers to the accession numbers from Flybase. “Gene symbol” refers to the gene symbol on Flybase. “Puncture fold change” refers to fold changes seen in expression values after puncture wounding relative to wild-type untreated values. “Trypsin fold change” refers to fold changes seen in gene expression values after trypsin puncture wounding relative to wild-type untreated values. “Highest fold change” refers to whether puncture or trypsin puncture wounding resulted in the highest fold change for the corresponding gene. #N/A indicates that the trypsin wounding treatment did not result in a significant fold change value (FDR>0.01). (PDF) [file pone.0061773.s009.pdf]

CG #	Gene symbol	Puncture fold change	Trypsin fold change	Highest fold change
CG18372	AttB	33.8	73.7	trypsin
CG15066	IM23	19.9	56.2	trypsin
CG10146	AttA	18.4	40.3	trypsin
CG18106	IM2	17.7	49.1	trypsin
CG15065	CG15065	17.6	44.2	trypsin
CG18108	IM1	16.6	38.9	trypsin
CG15231	IM4	16.0	28.0	trypsin
CG18067	CG18067	15.3	62.4	trypsin
CG16844	IM3	15.1	27.4	trypsin
CG15067	CG15067	14.9	37.9	trypsin
CG2444	CG2444	14.6	54.8	trypsin
CG16836	CG16836	11.4	33.8	trypsin
CG4740	AttC	10.7	44.1	trypsin
CG18279	IM10	10.6	18.4	trypsin
CG10810	Drs	10.0	39.0	trypsin
CG30080	CG30080	9.6	36.1	trypsin
CG18107	CG18107	9.4	38.1	trypsin
CG8175	Mtk	8.6	26.0	trypsin
CG7300	CG7300	8.1	7.1	puncture
CG10505	CG10505	7.8	4.8	puncture
CG6186	Tsf1	7.3	15.8	trypsin
CG7294	CG7294	6.8	24.2	trypsin
CG15068	CG15068	6.5	11.4	trypsin
CG13224	Cpr47Eb	6.4	29.8	trypsin
CG10794	DptB	6.0	7.5	trypsin
CG11668	CG11668	5.9	12.9	trypsin
CG14186	CG14186	5.7	9.3	trypsin
CG9649	CG9649	5.5	19.2	trypsin
CG2217	CG2217	5.5	13.9	trypsin
CG13422	CG13422	5.4	24.4	trypsin
CG14567	CG14567	5.3	45.9	trypsin
CG4381	GstD3	5.2	6.7	trypsin
CG5778	CG5778	5.1	19.9	trypsin
CG6188	CG6188	5.1	3.5	puncture
CG1864	Hr38	5.0	8.1	trypsin
CG11842	CG11842	5.0	16.8	trypsin
CG30091	CG30091	4.9	10.4	trypsin
CG16713	CG16713	4.6	10.1	trypsin
CG6023	CG6023	4.6	7.9	trypsin
CG9989	CG9989	4.6	7.2	trypsin
CG14059	CG14059	4.3	16.9	trypsin
CG4269	CG4269	4.2	6.1	trypsin
CG9451	CG9451	4.2	7.1	trypsin
CG14419	CG14419	4.0	2.6	puncture
CG1365	CecA1	3.9	2.5	puncture
CG14866	CG14866	3.8	14.9	trypsin
CG33192	MtnD	3.8	3.9	trypsin
CG5791	CG5791	3.8	12.4	trypsin
CG32241	CG32241	3.7	4.2	trypsin
CG1373	CecC	3.6	2.1	puncture
CG2045	Ser7	3.5	6.8	trypsin
CG1689	lz	3.5	14.0	trypsin
CG4316	Sb	3.5	7.7	trypsin
CG13659	CG13659	3.2	4.9	trypsin
CG15675	CG15675	3.2	4.0	trypsin
CG17107	CG17107	3.2	14.6	trypsin
CG4437	PGRP-LF	3.2	5.6	trypsin
CG5493	CG5493	3.2	7.2	trypsin
CG5157	CG5157	3.2	2.5	puncture
CG7459	Ctr1B	3.1	1.9	puncture
CG18349	Cpr67Fa2	3.1	#N/A	#N/A
CG2914	Ets21C	3.1	5.0	trypsin
CG15282	CG15282	3.1	6.9	trypsin
CG11992	Rel	3.1	6.4	trypsin
CG15678	CG15678	3.1	5.3	trypsin
CG34454	CG34454	3.1	21.9	trypsin
CG31769	CG31769	3.0	5.8	trypsin
CG13226	CG13226	3.0	11.9	trypsin
CG10842	Cyp4p1	3.0	5.1	trypsin
CG9733	CG9733	3.0	18.9	trypsin
CG11086	Gadd45	2.9	6.6	trypsin
CG3669	CG3669	2.9	6.7	trypsin
CG16704	CG16704	2.9	11.5	trypsin
CG8216	CG8216	2.9	4.3	trypsin
CG32185	CG32185	2.8	#N/A	#N/A
CG12868	CG12868	2.8	3.5	trypsin
CG15394	CG15394	2.8	14.7	trypsin
CG1851	Ady43A	2.7	9.3	trypsin
CG5993	os	2.7	4.3	trypsin
CG13699	CG13699	2.7	17.3	trypsin
CG4371	GstD7	2.7	7.6	trypsin
CG4998	CG4998	2.7	7.7	trypsin
CG7432	CG7432	2.7	21.1	trypsin
CG11450	net	2.7	#N/A	#N/A
CG1148	Osi2	2.7	14.7	trypsin
CG7941	Cpr67Fa1	2.6	#N/A	#N/A
CG1857	nec	2.6	6.2	trypsin
CG9438	Cyp6a2	2.6	2.5	puncture

CG30154	CG30154	2.6	5.9	trypsin
CG5162	CG5162	2.6	3.4	trypsin
CG9452	CG9452	2.6	4.6	trypsin
CG5391	CG5391	2.6	79.1	trypsin
CG9077	Cpr47Ec	2.6	23.4	trypsin
CG31370	CG31370	2.5	4.3	trypsin
CG8738	CG8738	2.5	3.8	trypsin
CG15279	CG15279	2.5	5.0	trypsin
CG6553	CG6553	2.5	9.5	trypsin
CG3540	Cyp4d14	2.5	#N/A	#N/A
CG6639	CG6639	2.5	5.5	trypsin
CG14695	CG14695	2.5	2.9	trypsin
CG9811	Rgk1	2.5	3.7	trypsin
CG4577	CG4577	2.5	3.9	trypsin
CG8170	CG8170	2.5	12.4	trypsin
CG4927	CG4927	2.5	4.0	trypsin
CG14606	CG14606	2.4	3.5	trypsin
CG9312	CG9312	2.4	8.9	trypsin
CG31288	CG31288	2.4	1.9	puncture
CG34165	CG34165	2.4	3.3	trypsin
CG33468	CG33468	2.4	3.7	trypsin
CG3666	Tsf3	2.4	9.9	trypsin
CG8620	CG8620	2.4	7.8	trypsin
CG10550	CG10550	2.4	2.9	trypsin
CG4026	IP3K1	2.3	2.2	puncture
CG10513	CG10513	2.3	#N/A	#N/A
CG30026	CG30026	2.3	3.7	trypsin
CG2065	CG2065	2.3	2.5	trypsin
CG17836	CG17836	2.3	2.7	trypsin
CG9134	CG9134	2.3	#N/A	#N/A
CG30151	CG30151	2.2	6.0	trypsin
CG33542	upd3	2.2	4.7	trypsin
CG6908	CG6908	2.2	#N/A	#N/A
CG13323	CG13323	2.2	5.9	trypsin
CG12763	Dpt	2.2	#N/A	#N/A
CG11012	Ugt37a1	2.2	4.4	trypsin
CG15102	Jheh2	2.2	#N/A	#N/A
CG9572	CG9572	2.2	6.1	trypsin
CG31300	CG31300	2.2	1.8	puncture
CG33462	CG33462	2.2	4.5	trypsin
CG31326	CG31326	2.2	6.8	trypsin
CG18641	CG18641	2.2	9.6	trypsin
CG5639	CG5639	2.2	10.6	trypsin
CG9360	CG9360	2.2	2.0	puncture
CG7539	Edg91	2.2	12.3	trypsin
CG15515	CG15515	2.1	2.3	trypsin
CG15784	CG15784	2.1	5.1	trypsin
CG15293	CG15293	2.1	3.6	trypsin
CG5550	CG5550	2.1	16.4	trypsin
CG13641	CG13641	2.1	2.2	trypsin
CG17044	yellow-e2	2.1	8.0	trypsin
CG10247	Cyp6a21	2.1	2.0	puncture
CG14219	CG14219	2.1	4.3	trypsin
CG7219	CG7219	2.1	10.3	trypsin
CG4821	Tequila	2.1	3.9	trypsin
CG10363	TepIV	2.1	4.2	trypsin
CG14495	CG14495	2.1	2.4	trypsin
CG10560	CG10560	2.1	3.3	trypsin
CG11709	PGRP-SA	2.0	6.2	trypsin
CG10211	CG10211	2.0	4.0	trypsin
CG7272	CG7272	2.0	1.8	puncture
CG8502	Cpr49Ac	2.0	5.9	trypsin
CG3962	Keap1	2.0	3.3	trypsin
CG6658	Ugt86Di	2.0	#N/A	#N/A
CG11878	CG11878	2.0	2.5	trypsin
CG33329	Sp212	2.0	7.6	trypsin
CG17524	GstE3	2.0	2.8	trypsin
CG13946	CG13946	2.0	#N/A	#N/A
CG16705	SPE	2.0	4.1	trypsin
CG13325	CG13325	2.0	3.6	trypsin
CG10245	Cyp6a20	2.0	2.4	trypsin
CG4432	PGRP-LC	2.0	2.5	trypsin
CG1367	CecA2	2.0	#N/A	#N/A
CG4739	Ugt86Dc	2.0	#N/A	#N/A
CG2060	Cyp4e2	2.0	#N/A	#N/A
CG32244	CG32244	2.0	#N/A	#N/A
CG17191	CG17191	2.0	5.6	trypsin
CG14680	Cyp12e1	1.9	2.0	trypsin
CG9616	CG9616	1.9	2.9	trypsin
CG9498	CG9498	1.9	4.1	trypsin
CG31436	CG31436	1.9	2.2	trypsin
CG6113	CG6113	1.9	#N/A	#N/A
CG5327	CG5327	1.9	3.3	trypsin
CG15695	CG15695	1.9	#N/A	#N/A
CG6955	Lcp65Ad	1.9	#N/A	#N/A
CG10045	GstD1	1.9	3.9	trypsin
CG14326	CG14326	1.9	22.8	trypsin
CG5008	GNBP3	1.9	3.3	trypsin
CG11395	CG11395	1.9	2.5	trypsin
CG4607	CG4607	1.9	2.3	trypsin
CG2056	spirit	1.9	3.5	trypsin
CG8317	CG8317	1.9	2.0	trypsin

CG3036	CG3036	1.9	2.5	trypsin
CG34035	CG34035	1.9	2.4	trypsin
CG8160	CG8160	1.9	1.9	trypsin
CG15046	CG15046	1.9	3.4	trypsin
CG4120	Cyp12c1	1.9	#N/A	#N/A
CG13780	Pvf2	1.9	1.9	trypsin
CG8205	fus	1.9	1.9	trypsin
CG13324	CG13324	1.9	10.1	trypsin
CG13862	CG13862	1.9	7.3	trypsin
CG10337	CG10337	1.9	4.6	trypsin
CG14704	PGRP-LB	1.8	3.5	trypsin
CG4847	CG4847	1.8	4.0	trypsin
CG3672	Cpr67B	1.8	6.7	trypsin
CG9441	Pu	1.8	3.5	trypsin
CG6283	CG6283	1.8	13.1	trypsin
CG1583	GIIIspla2	1.8	2.5	trypsin
CG16978	CG16978	1.8	#N/A	#N/A
CG11425	CG11425	1.8	3.6	trypsin
CG4182	yellow-c	1.8	5.7	trypsin
CG9453	Spn4	1.8	3.0	trypsin
CG33246	Ste:CG33246	1.8	#N/A	#N/A
CG41277	CG41277	1.8	2.4	trypsin
CG33346	CG33346	1.8	2.1	trypsin
CG3066	Sp7	1.8	3.4	trypsin
CG7450	CrebA	1.8	2.6	trypsin
CG4942	CG4942	1.8	2.4	trypsin
CG14275	CG14275	1.8	2.2	trypsin
CG16712	CG16712	1.8	2.8	trypsin
CG31104	CG31104	1.8	#N/A	#N/A
CG3597	CG3597	1.8	#N/A	#N/A
