# Supplementary material for: Serine Proteolytic Pathway Activation Reveals an Expanded Ensemble of Wound Response Genes in Drosophila
Source: PLoS One. 2013 Apr 24;8(4):e61773. doi: 10.1371/journal.pone.0061773 (PMC3634835; doi:10.1371/journal.pone.0061773)
Supplement: Text S1 — Statistical and Bioinformatical Analyses of Microarray Data. Below is a detailed explanation of how statistically significant genes from the Drosophila microarray sets were determined. (DOC) [file pone.0061773.s010.doc]

**Text S1. Statistical and Bioinformatical Analyses of Microarray Data.**

Statistical analyses of the microarray experiments are composed of three steps: 1) normalization of microarray data, 2) sorting genes according to interest, and 3) statistical analyses of Gene Ontology terms over-represented in the sorted list of genes.

**1) Normalization of microarray data.**

The Agilent Feature Extraction Software (v10.5) provides high quality expression level reports for Agilent microarrays. Nevertheless, these data do need to be normalized in order to remove subtle biases due to variations in hybridization conditions or manufacturing. We normalized all samples simultaneously using the multiple-*loess* technique described elsewhere (Sasik et al. 2004).

**2) Sorting genes according to significance.**

In designing the interest statistic we borrowed ideas from Cole et al. (2003) and their software package Focus. The interest statistic reflects the understanding that a gene with a greater fold change than another gene (in terms of absolute value) is likely to be more interesting and informative. For the purposes of false discovery rate (FDR) calculation, for each biological comparison, control and treatment samples were paired into two control-treatment pairs. Genes were ranked for each pair according to the log2 ratio of expression levels, and the rank-sum statistic was calculated for each gene. FDR for each gene was calculated exactly as the fraction of genes in the null model (defined as having uncorrelated ranks among the replicates) whose rank-sum statistic is as or more extreme than the one observed (Bradley and Gupta, 2002). Genes were then sorted and ranked according to their FDR.

**3) Statistical analyses of Gene Ontology terms.**

The integrated software platform Expander 5.0 (EXPression Analyzer and DisplayER) was used to test for enrichment of functionally related genes based on gene characteristics recorded in Gene Ontology (GO). This program is available for academic use at [http://acgt.](http://acgt.cs.tau.ac.il/expander)cs[.tau.](http://acgt.cs.tau.ac.il/expander)ac[.il/](http://acgt.cs.tau.ac.il/expander)expander. Tabular text files of statistically significant genes (FDR<0.01) for each time point and wounding treatment were uploaded into Expander’s TANGO (Tool for ANalsysis of GO enrichments) feature. TANGO looks for a statistically significant large intersection between the uploaded tabular text file genes and sets of genes annotated with some function. The standard statistical tests for enrichment of a group of genes annotated with a particular function employs a combination of the hypergeometic distribution and p-value generation. TANGO uses these two tests to identify significant enrichments (p<0.05) (Ulitsky et al. 2010).

**SUPPLEMENTAL REFERENCES**

Cole SW, Galic Z, Zack JA (2003) Controlling false-negative errors in microarray differential expression analysis: a PRIM approach. Bioinformatics 19(14): 1808–1816.

Sásik R, Woelk CH, Corbeil J (2004) Microarray truths and consequences. J Mol Endocrinol 33: 1–9.

Ulitsky, A. Maron-Katz, S. Shavit, D. Sagir, C. Linhart, R. Elkon, A. Tanay, R. Sharan, Y. Shiloh and R. Shamir (2010) Expander: from expression microarrays to networks and functions. Nat Protoc 5(2): 303-322

Bradley DM, Gupta CR (2002) On the distribution of the sum of n non-identically distributed uniform random variables. Ann Inst Stat Math 54(3): 689–700
